# *E. coli* surface display of streptavidin for directed evolution of an allylic deallylase†
†Electronic supplementary information (ESI) available. See DOI: 10.1039/c8sc00484f


**DOI:** 10.1039/c8sc00484f

**Published:** 2018-05-24

**Authors:** Tillmann Heinisch, Fabian Schwizer, Brett Garabedian, Eszter Csibra, Markus Jeschek, Jaicy Vallapurackal, Vitor B. Pinheiro, Philippe Marlière, Sven Panke, Thomas R. Ward

**Affiliations:** a Department of Chemistry , University of Basel , Mattenstrasse 24a , Basel CH-4002 , Switzerland . Email: thomas.ward@unibas.ch; b Institute of Structural and Molecular Biology , University College London , Gower Street , London , WC1E 6BT , UK; c Department of Biosystems Science and Engineering , ETH Zurich , Mattenstrasse 26 , Basel CH-4058 , Switzerland; d Heurisko USA Inc. , Delaware , USA

## Abstract

Artificial metalloenzymes (ArMs hereafter) combine attractive features of both homogeneous catalysts and enzymes and offer the potential to implement new-to-nature reactions in living organisms. Herein we present an *E. coli* surface display platform for streptavidin (Sav hereafter) relying on an Lpp-OmpA anchor. The system was used for the high throughput screening of a bioorthogonal CpRu-based artificial deallylase (ADAse) that uncages an allylcarbamate-protected aminocoumarin **1**. Two rounds of directed evolution afforded the double mutant S112M–K121A that displayed a 36-fold increase in surface activity *vs.* cellular background and a 5.7-fold increased *in vitro* activity compared to the wild type enzyme. The crystal structure of the best ADAse reveals the importance of mutation S112M to stabilize the cofactor conformation inside the protein.

## Abstract 2

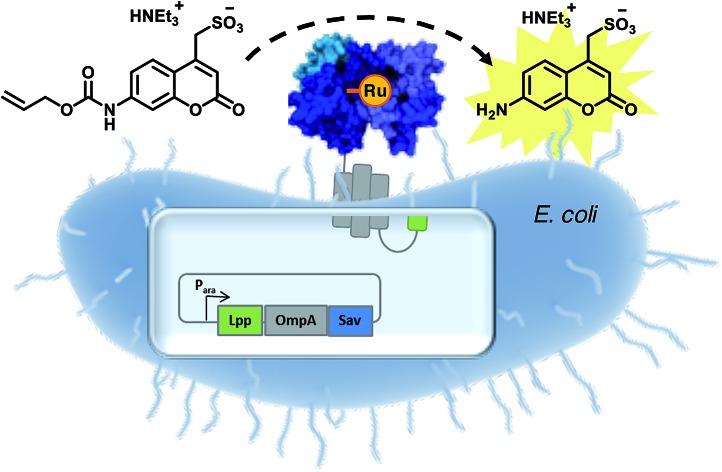
An artificial deallylase is constituted on the *E. coli* surface and genetically optimized for the deprotection of caged aminocoumarin.

## Introduction

A new generation of green, sustainable and biocompatible catalysts is a prerequisite to produce the fine chemicals and complex drugs of the future.^1^ ArMs consist of an organometallic moiety anchored within a protein scaffold thereby combining some of the most attractive features of both homogeneous catalysis and biocatalysis. In the last two decades, numerous reports have highlighted the potential of such biohybrid catalysts: (i) to catalyze new-to-nature transformations in water at room temperature, (ii) the possibility to improve the ArM's performance *via* chemo-genetic or directed evolution optimization schemes, and (iii) their integration in enzymatic cascades.^2^–^5^ Various strategies have been pursued toward the assembly of ArMs either *via* repurposing of natural metalloproteins or by anchoring a synthetic metal cofactor within a protein scaffold.^6^–^11^ Covalent-, dative- and supramolecular anchoring strategies have all been pursued with varying degrees of success.

The design and optimization process is most versatile when a combination of (i) structure-based cofactor design, (ii) *in silico* screening^12^,^13^ and (iii) directed evolution schemes are applied. Directed evolution offers virtually infinite sampling of protein space. In reality however, only a small fraction of the protein sequence space can be assessed due to the limited throughput of the screen.

The biotin-streptavidin technology ranks amongst the most versatile platforms for the generation of ArMs.^8^,^14^,^15^ In the past, the throughput of Sav-based ArMs screens has been limited by the presence of cellular inhibitors (mainly glutathione), poisoning the transition metal, thus requiring screening with purified Sav samples. Two strategies have been introduced to overcome this challenge: (i) addition of thiol scavengers to cellular extracts^16^–^18^ and (ii) Sav secretion into *E. coli*'s periplasm that contains low concentrations of reduced thiols.^19^ As a proof-of-principle, we demonstrated that a Sav-based artificial metalloenzyme for ring-closing metathesis could be assembled and evolved in the periplasm of *E. coli*.^19^ Integration of new-to-nature reactions in a microbial organism offers fascinating perspectives in chemical- and synthetic biology. Sav-based ArMs assembled in the periplasm however require the passive diffusion of the biotinylated metal cofactor across the *E. coli* outer membrane.

**Fig. 1:**
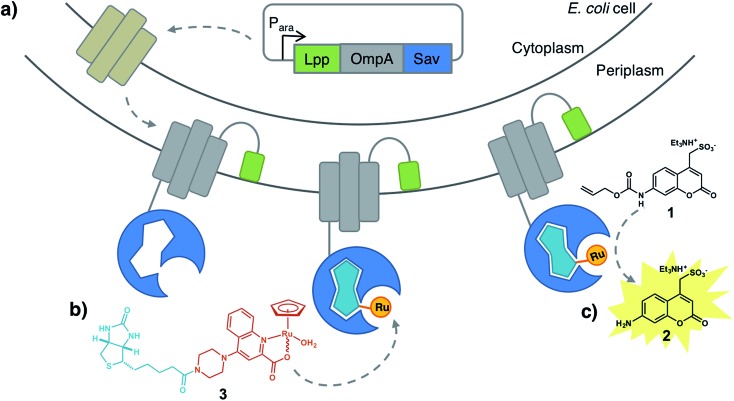
Artificial metalloenzyme displayed on the *E. coli* outer membrane. (a) Streptavidin is fused to the C-terminus of an Lpp-OmpA fragment. Induction of Lpp-OmpA-Sav expression in the cytosol is followed by secretion into the periplasmic space and anchoring to the outer membrane. For simplicity only one Sav monomer is displayed. (b) A biotinylated ruthenium catalyst [CpRu(QA-Biot)(OH_2_)] **3** binds to Sav to afford an artificial deallylase. (c) A fluorescent aminocoumarin **2** is uncaged in the presence of ADAse on the *E. coli* surface.

To circumvent this limitation, we reasoned that an *E. coli* surface display might offer an attractive means to assemble an ArM while maintaining the critical phenotype–genotype linkage to allow evolving the biohybrid catalyst. Towards this goal, we identified the following challenges: (i) create a high-performance bioorthogonal ArM, (ii) identify a fluorogenic substrate that is uncaged in the presence of low catalyst concentrations (∼1 μM) in the presence of a living cell, (iii) develop a platform to display Sav on *E. coli*'s outer membrane, (iv) demonstrate binding of the biotinylated cofactor on surface-displayed Sav and (v) develop a fluorescent assay, amenable to high-throughput screening (Fig. 1).

## Results and discussions

Building on the reports of Meggers, Wender and Mascareñas, we selected the CpRu(2-quinolinecarboxylate)-catalyzed uncaging of an allylcarbamate-protected substrate as a model reaction.^20^–^23^ This system is particularly attractive as the Ru-catalyzed allylic substitution was shown to (i) tolerate a cellular environment, (ii) remain active even at very low catalyst concentration, (iii) require no additional external nucleophile (either water or thiols (*i.e.* glutathione) act as nucleophile to uncage the substrate *in vivo*) and (iv) be bioorthogonal.

We selected the water-soluble fluorogenic substrate 7-allylcarbamate-4-sulfonyl coumarin (**1** hereafter) to demonstrate the feasibility of surface-displayed ArMs based on the biotin-streptavidin technology (Fig. 1). We synthesized the biotinylated quinoline ligand QA-Biot (see ESI† for details). The activity of [CpRu(QA-Biot)(OH_2_)] **3**, obtained upon *in situ* complexation of the biotinylated ligand to [CpRu(CH_3_CN)_3_]^+^, was evaluated for the uncaging of substrate **1** (Fig. 1b, c and S1†). The *in vitro* reaction performed best in phosphate-buffered saline at pH 7.4 (Fig. S11†) producing 7 turnover numbers (TON) after 30 h (Fig. 3b and S13b†). The Sav-embedded catalyst [CpRu(QA-Biot)(OH_2_)] **3**·WT Sav (ADAse) performed 3.7-fold better (26 TON) than the free cofactor [CpRu(QA-Biot)(OH_2_)] **3** at 0.2 mol% loading in WT Sav. The protein was applied in 2-fold excess *vs.* cofactor **3** with respect to the free biotin binding sites (*i.e.* 1 μM [CpRu(QA-Biot)(OH_2_)] **3** and 0.5 μM homotetrameric Sav). The release of aminocoumarin **2** could be detected in the presence of 1 μM ArM. Virtually no background activity was observed in the absence of [CpRu(QA-Biot)(OH_2_)] **3** at pH 7.4.

### Sav surface display

Having identified reaction conditions suitable for the *in vitro* aminocoumarin **2** formation with an ADAse, we set out to develop a Sav-display platform on the *E. coli* outer membrane.^24^,^25^ Initial efforts focused on the fusion construct between Sav with autotransporter AIDA as reported by Pyun and coworkers.^26^ As the expression levels proved rather low, we turned our attention to the Lpp-OmpA anchor initially reported by Georgiou *et al.*^27^–^29^

**Fig. 2:**
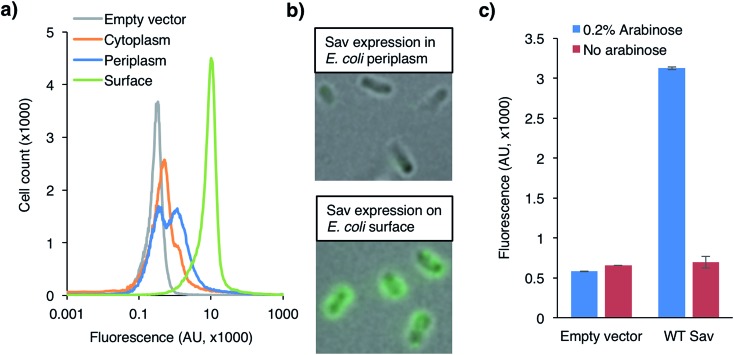
(a) Flow cytometry of immuno-stained *E. coli* cells expressing Sav in the cytoplasm (orange), periplasm (blue) or on the surface (green) compared to the empty vector control (grey). Cells were labeled with a primary mouse anti-Sav-antibody followed by a fluorescently-labeled secondary goat-anti-mouse antibody. (b) Fluorescence microscopy of immuno-stained *E. coli* cells from (a). (c) Uncaging of fluorogenic substrate **1** with WT ADAse on *E. coli*'s surface in absence (red) and presence (blue) of 0.2% l-arabinose that induces the overexpression and display of WT Sav on *E. coli*'s outer surface.

This anchor consists of the truncated *E. coli* lipoprotein Lpp (residues 1–9) fused to the first five β-strands of outer membrane protein OmpA (residues 46–159). Sav was fused to the C-terminus of OmpA and directed towards the extracellular space (Fig. S14 and S15†). Successful integration of Sav on the outer membrane was highlighted by staining the cells with a primary mouse-anti Sav antibody, followed by labeling with a secondary fluorescent antibody. The samples were analyzed by flow cytometry (Fig. 2a) and fluorescence microscopy (Fig. 2b). Flow cytometry revealed a marked increase in fluorescence in the presence of cells with Sav displayed on the outer membrane *vs.* cells with Sav localized either in the cytoplasm or in the periplasm. As we anticipated that antibodies would not cross the outer- or inner membrane, fluorescence detected as a result of antibody staining strongly supports that Sav is indeed displayed on the outer-membrane and oriented towards the extracellular space. Minimally biphasic fluorescence was however also observed for the periplasmic Sav sample (*i.e.* using the OmpA Sav construct that secretes Sav to *E. coli*'s periplasm^19^), suggesting partial antibody migration through the permeabilized outer membrane. Similar effects have been reported in other Lpp-OmpA-labeling studies.^30^ Fluorescence microscopy revealed *E. coli* fluorescence labeling only in the presence of cells with Sav displayed on the surface. Monitoring surface Sav expression with a fluorescently-labeled biotin (Biot-ATTO565) yielded very similar results (Fig. S16†). Although the oligomeric state of Sav was not assessed, we hypothesize that it is displayed as a homotetramer as demonstrated for other surface-displayed oligomeric proteins.^31^,^32^

### ADAse with surface-displayed Sav

Next, we tested the ADAse's activity with the surface-displayed biohybrid catalyst. The Sav isoforms were expressed in *E. coli* cells at 25 °C for 4 h in a 96-well plate. The cells were harvested, the pellets were washed with PBS and resuspended in the cofactor buffer (2 μM **3** in PBS, pH 7.4). After incubation on ice for 30 min, the cells were spun-down, the pellets were washed twice with PBS and resuspended in the substrate buffer (500 μM **1** in PBS, pH 7.4) and the cells were incubated at 30 °C for 16 h. The formation of aminocoumarin **2** was quantified by fluorescence (excitation 395 nm, emission 460 nm; see ESI†). To our delight, the surface-displayed ArMs revealed ADAse activity. The ADAse [CpRu(QA-Biot)(OH_2_)] **3**·WT Sav had a 1.5-fold increase over cellular background (Fig. S18†). Importantly, catalysis for 16 h did not stall the viability of *E. coli* cells as highlighted by cell growth on LB-agar plates (Fig. S19†).

Taken together, fluorescence labeling and catalysis experiments with Lpp-OmpA-Sav demonstrate that (i) streptavidin is expressed and displayed on the outer membrane of *E. coli*, (ii) the surface-displayed Sav maintains its biotin-binding activity towards both Biot-ATTO565 as well as a biotinylated ruthenium complex [CpRu(QA-Biot)(OH_2_)] **3**, (iii) the surface-displayed ADAse is functional and (iv) cells are viable after catalysis.

**Fig. 3:**
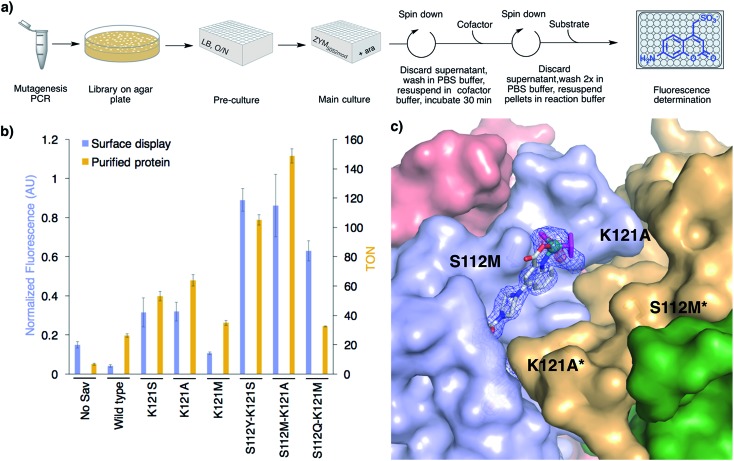
High-throughput screening on *E. coli*'s surface to optimize ADAse activity. (a) 96-well plate screen. (b) Summary of the results from the directed evolution of ADAses on *E. coli*'s surface (blue bars) and catalysis with purified Sav mutants (gold bars) identified using the surface-display screen. Reaction conditions with purified Sav samples: 500 μM substrate **1**, 0.2 mol% cofactor **3**, 0.4 mol% Sav_monomer_, 30 h, RT. (c) Crystal structure of an evolved ADAse [CpRu(QA-Biot)(OH_2_)] **3**·Sav S112M–K121A (PDB ; 6FH8). The protein is displayed as solvent-accessible surface and the cofactor and residues S112M and K121A as stick models. The cofactor **3** is contoured with electron density of a 2FoFc map in blue (1*σ*) and an anomalous dispersion density in red (4*σ*). The ruthenium is displayed as a turquoise sphere. The ligands highlighted in magenta were not included in the structure refinement as no electron density could be unambiguously identified. Only one cofactor within the bis-biotin binding vestibule is depicted for clarity.

With a 96-well plate fluorimetric screen at hand (Fig. 3a), we set out to genetically evolve the surface-displayed ADAse. Previous Sav-ArM evolution campaigns highlighted the potential of iterative site saturation mutagenesis at positions S112 and K121 which lie in the immediate proximity of the catalytic moiety.^17^–^19^ We thus generated a saturation mutagenesis library at position Sav K121**X** (see ESI†).^33^ Several Sav mutants displayed increased surface ADAse activity *vs.* WT Sav: Sav K121S (7.2-fold), Sav K121A (6.4-fold) and Sav K121M (2.6-fold) (Fig. S18†). Removal of the basic lysine in position K121 thus appears to be beneficial. The three best Sav single mutants (*i.e.* K121S, K121A and K121M) identified in the surface-display screen were subjected to saturation mutagenesis at position S112 and the ADAse activity of the corresponding three libraries (*i.e.* Sav S112**X**–K121S, Sav S112**X**–K121A and Sav S112**X**–K121M) was analyzed by fluorescence using the surface-displayed Sav double mutants in a 96 well plate format. For each of these three libraries, this straightforward screen allowed us to identify double mutants with increased surface-displayed ADAse activity: the two best second generation mutants Sav S112Y–K121S and S112M–K121A yielded 25- and 24-fold increased activity *vs.* WT ADAse, respectively. The best hit resulting from library S112X–K121M was S112Q–K121M affording a 17-fold improved activity *vs.* the WT ADAse, respectively (Fig. 3b and S18†). We conclude that bulky and polar residues at position Sav S112 lead to improved ADAse activity. Importantly, the activity of the best surface-displayed ADAse was 37-fold larger *vs.* the empty vector control. A linear reaction rate of surface-displayed ADAse for at least 16 h demonstrates the longevity of this hybrid system (Fig. S13a†). To investigate potential cofactor accumulation inside cells and degradation of the surface-displayed ADAse, we incubated cells carrying either an empty plasmid or the Sav S112Y–K121S plasmid with excess cofactor **3** for 10, 30, 60 and 120 min before washing. The ADAse activity of the cells carrying the empty vector did not change even following a two hour incubation with the [CpRu(QA-Biot)(OH_2_)] **3** cofactor (Fig. S20†). This suggests that the cofactor does not accumulate in the cells over time. Gratifyingly, the S112Y–K121S ADAse maintained its activity even after two hours incubation. This supports our hypothesis that no detectable surface-displayed ADAse degradation takes place.

### Catalysis with purified ADAses

Next, we expressed the best single (K121S, K121A and K121M) and three best double Sav mutants (*i.e.* Sav S112Y–K121S, Sav S112M–K121A and Sav S112Q–K121M) in the cytoplasm, and purified them by affinity chromatography (see ESI†). The *in vitro* activity ranking of the best Sav single mutants slightly changed to K121A > K121S > K121M and the activity improvement *vs.* WT was less pronounced than in the surface-display screen (2.4-, 2.0- and 1.3-fold, respectively, Fig. 3b). The double mutant S112M–K121A displayed the highest activity *in vitro*. The TON increase *vs.* WT was 5.7-fold. The purified ADAses displayed a linear reaction rate for 30 h, which is similar to the corresponding surface-displayed enzymes (Fig. S13b). To correlate the activities of the evolved ADAses in purified form and on-cells, we determined the relative expression yield of surface-displayed Sav using Biot-ATTO565 staining followed by flow cytometry (Fig. S17). All cells with surface-displayed Sav mutants displayed a fluorescence between 5- and 9-fold higher than the empty vector control. Upon normalization of the on-cell activity for Sav expression, the order of the most active mutants remained the same as determined without this normalization procedure (Fig. 3b and S18†). Accordingly, the best surface-displayed ADAses [CpRu(QA-Biot)(OH_2_)] **3**·Sav S112Y–K121S and **3**·Sav S112M–K121A are 22- and 21-fold more active than the WT ADAse.

### X-ray structure

In order to elucidate the localization of [CpRu(QA-Biot)(OH_2_)] **3** within Sav, we sought to gain X-ray structural insight. To our delight, [CpRu(QA-Biot)(OH_2_)] **3**·Sav-S112M–K121A afforded high resolution diffraction data. The crystals were obtained by sitting drop vapor diffusion, followed by soaking with cofactor [CpRu(QA-Biot)(OH_2_)] **3** and flash freezing in liquid nitrogen (see ESI†). The [CpRu(QA-Biot)(OH_2_)] **3** cofactor was modeled with 100% occupancy within the Sav biotin binding site (Fig. 3c and S21†). The piperazine linker and the ruthenium carboxyquinoline moiety are localized in a single conformation within the biotin vestibule. The aromatic quinoline ring is stabilized by a methionine–π interaction with the neighboring S112M side chain.^34^ The aliphatic side chains of L124 and L124* (* indicates the neighboring Sav monomer) and the carbonyl moieties of residues K121A and S122 form a pocket that harbors the quinoline ligand. Spherically-shaped electron density around the ruthenium suggests that the cofactor may exist as a mixture of epimers (*i.e.* (*R*)- and (*S*)-Ru). The Cp and water ligands were thus not modeled due to the ambiguity of the electron density.

## Conclusions

We evolved an artificial allylic deallylase based on the biotin-streptavidin technology that catalyzes the uncaging of a fluorogenic allylcarbamate-protected coumarin **1**. The reaction proceeds smoothly even at 1 μM catalyst concentration. To streamline the directed evolution protocol, we implemented an *E. coli* outer membrane display using the Lpp-OmpA-Sav fusion construct. Amino-acid substitutions at positions S112 and K121 lead to a twenty-one-fold improvement in normalized surface ADAse activity (*i.e.* Sav S112M–K121A) *vs.* WT. The Sav mutations accelerating ADAse activity on the *E. coli* surface also have a beneficial effect on purified Sav samples *in vitro* yielding an up to 5.7-fold increased TON (Sav S112M–K121A) *vs.* WT. *E. coli* cells are viable after 16 h catalysis. X-ray crystallography of ADAse double mutant S112M–K121A highlights the localization of cofactor **3** within the biotin-binding vestibule. We anticipate that this straightforward protein-display strategy can readily be extended to significantly simplify the directed evolution of artificial metalloenzymes for *in vivo* synthetic biology applications.

## Conflicts of interest

The authors declare no competing financial interest.

## Supplementary Material

Supplementary information

## References

[cit1] Bornscheuer U. T., Huisman G. W., Kazlauskas R. J., Lutz S., Moore J. C., Robins K. (2012). Nature.

[cit2] Schwizer F., Okamoto Y., Heinisch T., Gu Y. F., Pellizzoni M. M., Lebrun V., Reuter R., Köhler V., Lewis J. C., Ward T. R. (2018). Chem. Rev..

[cit3] Yu F., Cangelosi V. M., Zastrow M. L., Tegoni M., Plegaria J. S., Tebo A. G., Mocny C. S., Ruckthong L., Qayyum H., Pecoraro V. L. (2014). Chem. Rev..

[cit4] Lewis J. C. (2013). ACS Catal..

[cit5] Ilie A., Reetz M. T. (2015). Isr. J. Chem..

[cit6] Brandenberg O. F., Fasan R., Arnold F. H. (2017). Curr. Opin. Biotechnol..

[cit7] Dydio P., Key H. M., Nazarenko A., Rha J. Y., Seyedkazemi V., Clark D. S., Hartwig J. F. (2016). Science.

[cit8] Heinisch T., Ward T. R. (2016). Acc. Chem. Res..

[cit9] Song W. J., Tezcan F. A. (2014). Science.

[cit10] Key H. M., Dydio P., Clark D. S., Hartwig J. F. (2016). Nature.

[cit11] Srivastava P., Yang H., Ellis-Guardiola K., Lewis J. C. (2015). Nat. Commun..

[cit12] Heinisch T., Pellizzoni M., Dürrenberger M., Tinberg C. E., Köhler V., Klehr J., Häussinger D., Baker D., Ward T. R. (2015). J. Am. Chem. Soc..

[cit13] Blomberg R., Kries H., Pinkas D. M., Mittl P. R. E., Grutter M. G., Privett H. K., Mayo S. L., Hilvert D. (2013). Nature.

[cit14] Reetz M. T., Peyralans J. J. P., Maichele A., Fu Y., Maywald M. (2006). Chem. Commun..

[cit15] Wilson M. E., Whitesides G. M. (1978). J. Am. Chem. Soc..

[cit16] Wilson Y. M., Dürrenberger M., Nogueira E. S., Ward T. R. (2014). J. Am. Chem. Soc..

[cit17] Mallin H., Hestericová M., Reuter R., Ward T. R. (2016). Nat. Protoc..

[cit18] Hestericová M., Heinisch T., Alonso-Cotchico L., Maréchal J. D., Vidossich P., Ward T. R. (2018). Angew. Chem., Int. Ed..

[cit19] Jeschek M., Reuter R., Heinisch T., Trindler C., Klehr J., Panke S., Ward T. R. (2016). Nature.

[cit20] Volker T., Dempwolff F., Graumann P. L., Meggers E. (2014). Angew. Chem., Int. Ed..

[cit21] Volker T., Meggers E. (2017). ChemBioChem.

[cit22] Hsu H. T., Trantow B. M., Waymouth R. M., Wender P. A. (2016). Bioconjugate Chemistry.

[cit23] Tomás-Gamasa M., Martínez-Calvo M., Couceiro J. R., Mascareñas J. L. (2016). Nat. Commun..

[cit24] Jeschek M., Panke S., Ward T. R. (2016). Methods Enzymol..

[cit25] Grimm A. R., Sauer D. F., Polen T., Zhu L., Hayashi T., Okuda J., Schwaneberg U. (2018). ACS Catal..

[cit26] Park M., Jose J., Thommes S., Kim J. I., Kang M. J., Pyun J. C. (2011). Enzyme Microb. Technol..

[cit27] Francisco J. A., Earhart C. F., Georgiou G. (1992). Proc. Natl. Acad. Sci. U. S. A..

[cit28] Yang C., Zhao Q., Liu Z., Li Q. Y., Qiao C. L., Mulchandani A., Chen W. (2008). Environ. Sci. Technol..

[cit29] Peschke T., Rabe K. S., Niemeyer C. M. (2017). Angew. Chem., Int. Ed..

[cit30] Stathopoulos C., Georgiou G., Earhart C. F. (1996). Appl. Microbiol. Biotechnol..

[cit31] Jose J., Bernhardt R., Hannemann F. (2001). ChemBioChem.

[cit32] Jose J., von Schwichow S. (2004). ChemBioChem.

[cit33] Kille S., Acevedo-Rocha C. G., Parra L. P., Zhang Z. G., Opperman D. J., Reetz M. T., Acevedo J. P. (2013). ACS Synth. Biol..

[cit34] Valley C. C., Cembran A., Perlmutter J. D., Lewis A. K., Labello N. P., Gao J., Sachs J. N. (2012). J. Biol. Chem..

