# Correction: *E. coli* surface display of streptavidin for directed evolution of an allylic deallylase

**DOI:** 10.1039/c9sc90139f

**Published:** 2019-06-28

**Authors:** Tillmann Heinisch, Fabian Schwizer, Brett Garabedian, Eszter Csibra, Markus Jeschek, Jaicy Vallapurackal, Vitor B. Pinheiro, Philippe Marlière, Sven Panke, Thomas R. Ward

**Affiliations:** a Department of Chemistry , University of Basel , Mattenstrasse 24a , Basel CH-4002 , Switzerland . Email: thomas.ward@unibas.ch; b Institute of Structural and Molecular Biology , University College London , Gower Street , London , WC1E 6BT , UK; c Department of Biosystems Science and Engineering , ETH Zurich , Mattenstrasse 26 , Basel CH-4058 , Switzerland; d Heurisko USA Inc. , Delaware , USA

## Abstract

Correction for ‘*E. coli* surface display of streptavidin for directed evolution of an allylic deallylase’ by Tillmann Heinisch *et al.*, *Chem. Sci.*, 2018, **9**, 5383–5388.



## 

In the original manuscript, funding support from the ERC (HNAepisome; grant number 336936) was mistakenly omitted from the acknowledgements section. The corrected acknowledgements section is given below:

TRW thanks the SNF (grant number grant 200020_162348) and the EU (ERC grant, the DrEAM) and SP and TRW thank the NCCR Molecular Systems Engineering for generous support. VBP and EC also acknowledge support from the ERC (HNAepisome; grant number 336936). Juliane Klehr is thanked for help with vector cloning, protein expression and purification, Dr Rosario Vanella for help with flow cytometry and fluorescence microscopy and Eleonore Schmidt for activity measurement of purified ADAse mutants.

The Royal Society of Chemistry apologises for these errors and any consequent inconvenience to authors and readers.

