# Supplementary material for: E. coli surface display of streptavidin for directed evolution of an allylic deallylase
Source: Chem Sci. 2018 May 24;9(24):5383–8. doi: 10.1039/c8sc00484f (PMC6048633; doi:10.1039/c8sc00484f)
Supplement: Supplementary file 1 [file SC-009-C8SC00484F-s001.pdf]

Electronic Supplementary Material (ESI) for Chemical Science.
This journal is © The Royal Society of Chemistry 2018

Supplementary Information

E. coli surface display of streptavidin for directed evolution of an allylic deallylase

Tillmann Heinisch,[1,+] Fabian Schwizer, [1,+] Brett Garabedian,[1] Eszter Csibra,[2] Markus Jeschek,[3] Jaicy Vallapurackal[1], Vitor B. Pinheiro,[2] Philippe Marlière,[4] Sven Panke,[2] Thomas R. Ward[1,]*

([+] authors contributed equally)

[1]Department of Chemistry, University of Basel, Mattenstrasse 24a, Basel CH-4002, Switzerland.
[2]Institute of Structural and Molecular Biology, University College London, Gower Street, London, WC1E 6BT, U.K.
[3]Department of Biosystems Science and Engineering, ETH Zurich, Mattenstrasse 26, Basel CH-4058, Switzerland.
[4]Heurisko USA Inc., Delaware, US.

1. Materials 3

2. Substrate **1** and cofactor **3** synthesis 4

3. *In vitro* deprotection of substrate **1** using ADAse 26

4. Cloning vector pBAD33_Lpp-OmpA-Sav for streptavidin display on *E. coli* surface 29

5. Expression of streptavidin displayed on *E. coli* surface 40

6. Labeling of surface-displayed streptavidin with a fluorescent antibody 41

7. Labeling of surface-displayed streptavidin with Biot-ATTO-565 42

8. Determination of the expression yield of surface-displayed Sav 43

9. Creation of site-saturation mutagenesis libraries using the 22-codon trick 45

10. 96-well plate activity assay using *E. coli* surface display of streptavidin isoforms 48

11. *E. coli* cell survival during surface-display catalysis 51

12. Investigation of the potential of biotinylated cofactor **3** to accumulate inside cells .. 52

13. Expression and purification of streptavidin mutants 53

14. Crystal structure determination of [CpRu(QA-Biot)(H_2O)] **3** · Sav-S112M-K121A 53

15. Literature 57

1. Materials

All commercially available chemicals were purchased from Sigma-Aldrich, ABCR, TCI Europe, Acros Organics, Alfa Aesar, Fluka, Fluorochem, Merck or Ukrorgsyntez Ltd. and used without further purification. Proteins for molecular biology were obtained from New England Biolabs (NEB). NMR spectra were measured on a 400 MHz and 500 MHz Bruker Advance spectrometer at room temperature and evaluated with MestReNova. Chemical shifts (δ) are listed in parts per million (ppm) and referenced to the residual solvent peaks. Scalar coupling (J) is reported in Hertz (Hz). HRMS analysis was performed on a Bruker maXis 4G mass spectrometer. Mass-spectral analysis of the expressed streptavidin mutants was performed on a Bruker Daltonics, ESI/micrOTOF MS. Fluorescence/Absorption spectroscopy was performed on a TECAN infinite M1000 Pro. DNA sequencing (Sanger cycle sequencing/capillary electrophoresis) was performed by Microsynth AG. Affinity column chromatography (purification of the expressed streptavidin mutants) was performed on an Äktaprime Plus chromatography system, using 2-iminobiotin sepharose column. Flow cytometry analysis was performed on an Attune NxT acoustic focusing cytometer (life technologies^{TM}).

2. Substrate **1** and cofactor **3** synthesis

The synthesis of the biotinylated quinoline ligand QA-Biot **10** was performed following a five-step procedure, starting from kynurenic acid **4** (Figure S1). The catalytically active ruthenium cofactor [CpRu(QA-Biot)(H_2O)] **3** was formed *in situ* and subsequently incorporated into streptavidin.

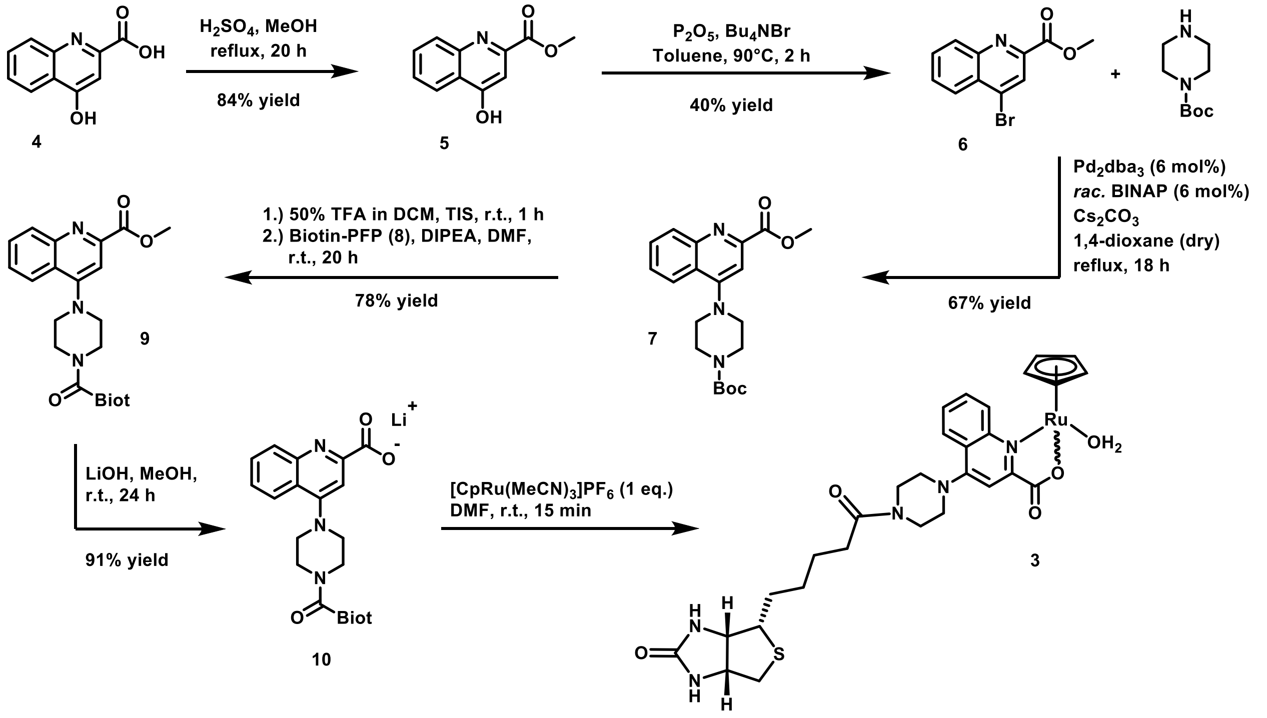

Figure S1: Synthesis of cofactor QA-Biot **10** and the corresponding Ru complex **3**.

Compound 5:

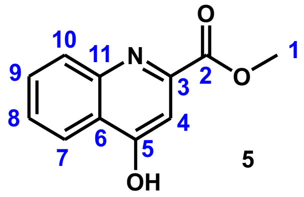

5

Kynurenic acid (**4**, 5.00 g, 26.4 mmol, 1.0 eq.) was suspended in dry methanol (50 ml, 39.6 g, 1240 mmol, 47 eq.). Concentrated sulfuric acid (3.00 ml, 5.50 g, 56.3 mmol, 2.1 eq.) was added, whereupon the mixture became clear. The solution was heated at reflux for 20 h under an N_2 atmosphere. The solution was evaporated to dryness, yielding a yellow oil. Water (100 ml) and saturated aqueous $NaHCO_3$ (100 ml) were added, whereupon an off-white solid precipitated. The solid was filtered, washed with diethyl ether (50 ml) and dried to yield the product as a white solid (**5**, 4.53 g, 22.3 mmol, 84% yield).

^{1}H NMR (400 MHz, DMSO-d_6, δ/ppm): 12.09 (s, 1H, **OH**), 8.08 (dd, *J* = 8.1, 1.5 Hz, 1H, **7**), 7.94 (d, *J* = 8.4 Hz, 1H, **10**), 7.71 (ddd, *J* = 8.5, 7.0, 1.6 Hz, 1H, **9**), 7.38 (ddd, *J* = 8.1, 7.0, 1.1 Hz, 1H, **8**), 6.66 (s, 1H, **4**), 3.96 (s, 3H, **1**). Solvents: Water (3.37), DMSO (2.50).

^{13}C NMR (101 MHz, DMSO-d_6, δ/ppm): 162.85 (1C, **2**), 140.45 (1C, **11**, extrapolated from HMBC spectrum), 132.48 (1C, **9**), 125.80 (1C, **6**), 124.59 (1C, **7**), 124.10 (1C, **8**), 120.00 (1C, **10**), 109.90 (1C, **4**), 53.46 (1C, **1**). Solvents: DMSO (40.15, 39.94, 39.73, 39.52, 39.31, 39.10, 38.89). The signals for the quaternary carbons **3** and **5** could not be resolved.

HRMS (ESI-MS, pos.) m/z: $[M+Na]^+$ calculated for $C_{11}H_9NO_3Na$: 226.0475, found: 226.0476.

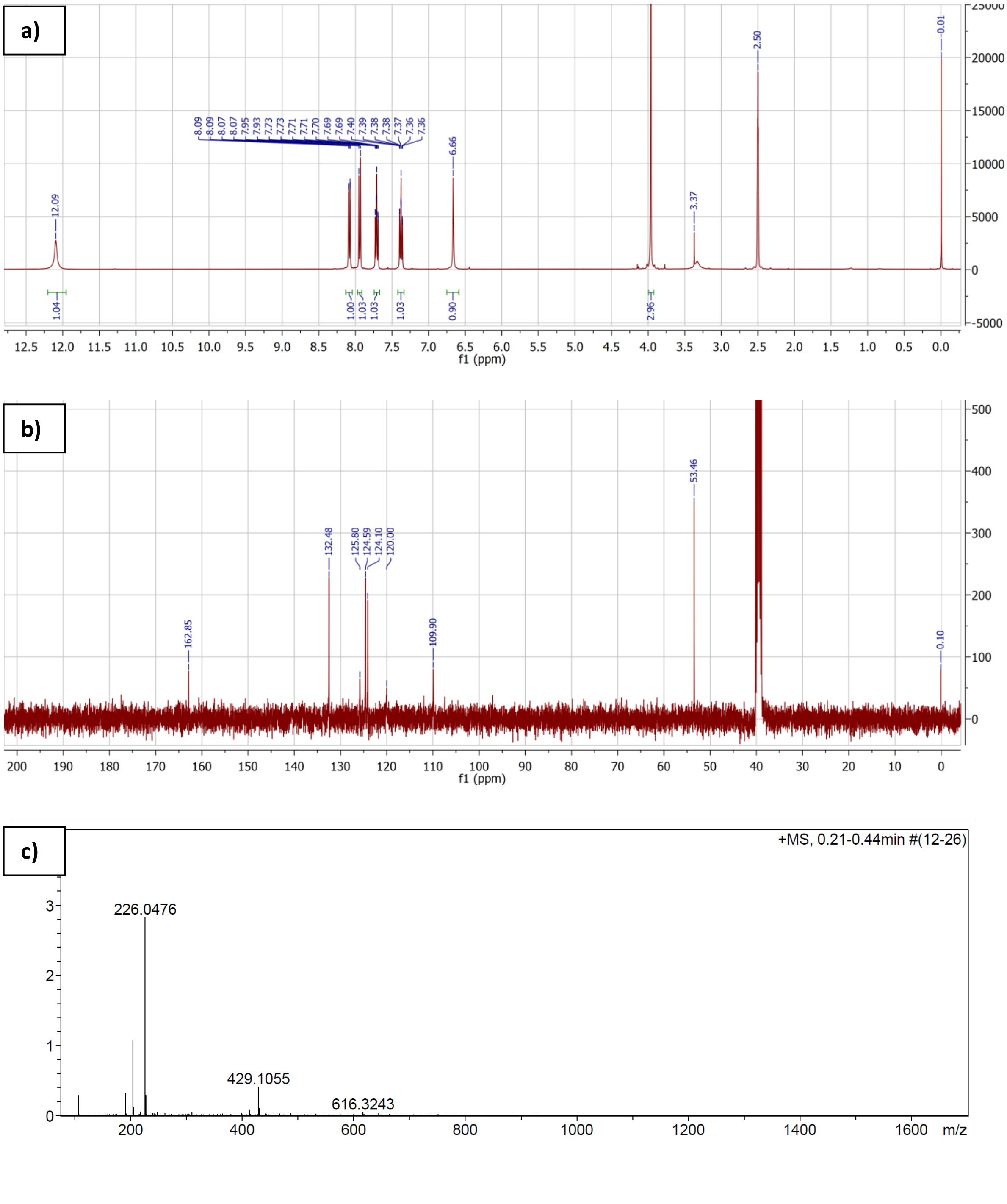

Figure S2: a) ^{1}H-NMR, b) ^{13}C-NMR and c) HRMS spectrum of compound **5**.

Compound 6:

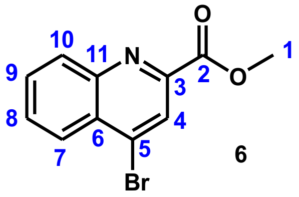

6

A mixture of methyl ester **5** (2.30 g, 11.3 mmol, 1.0 eq.), P_2O_5 (3.55 g, 24.9 mmol, 2.2 eq.) and Bu_4NBr (4.02 g, 12.4 mmol, 1.1 eq.) in toluene (80 ml) was heated at 90°C for 1 h with vigorous stirring. After cooling to room temperature, the upper-layer (i.e. toluene) was collected. The organic phase was washed with saturated $NaHCO_3$ (150 ml), brine (150 ml) and water (150 ml), dried over Na_2SO_4 and concentrated under reduced pressure to yield a yellow solid (**6**, 1.10 g, 4.12 mmol, 36% yield). The crude product was used for the next synthesis step without further purification.

^{1}H NMR (400 MHz, DMSO-d_6, δ/ppm): 8.38 (s, 1H, **4**), 8.23 (s (broad), 1H, **7 or 10**), 8.21 (s (broad), 1H, **7 or 10**), 8.01 – 7.94 (m, 1H, **8** or **9**), 7.94 – 7.88 (m, 1H, **8** or **9**), 3.97 (s, 3H, **1**). Solvents: Toluene (7.24, 7.17, 2.29), Water (3.35), DMSO (2.50).

^{13}C NMR (101 MHz, DMSO-d_6, δ/ppm): 164.17 (1C, **2**), 147.52 (1C), 147.30 (1C), 134.29 (1C), 131.80 (1C), 130.63 (1C), 130.60 (1C), 127.94 (1C), 126.31 (1C), 124.52 (1C), 52.90 (1C, **1**). Solvents: Toluene (137.32, 128.87, 128.18, 125.29, 21.03), DMSO (40.15, 39.94, 39.73, 39.52, 39.31, 39.10, 38.89). Impurities: 29.04, 23.37.

UPLC-MS (ESI-MS, pos.) *m/z*: $[M+H]^+$ calculated for $C_{12}H_8{}^{79}BrNO_2$: 265.98, found: 266.02; calculated for $C_{12}H_8{}^{81}BrNO_2$: 267.98, found: 267.97.

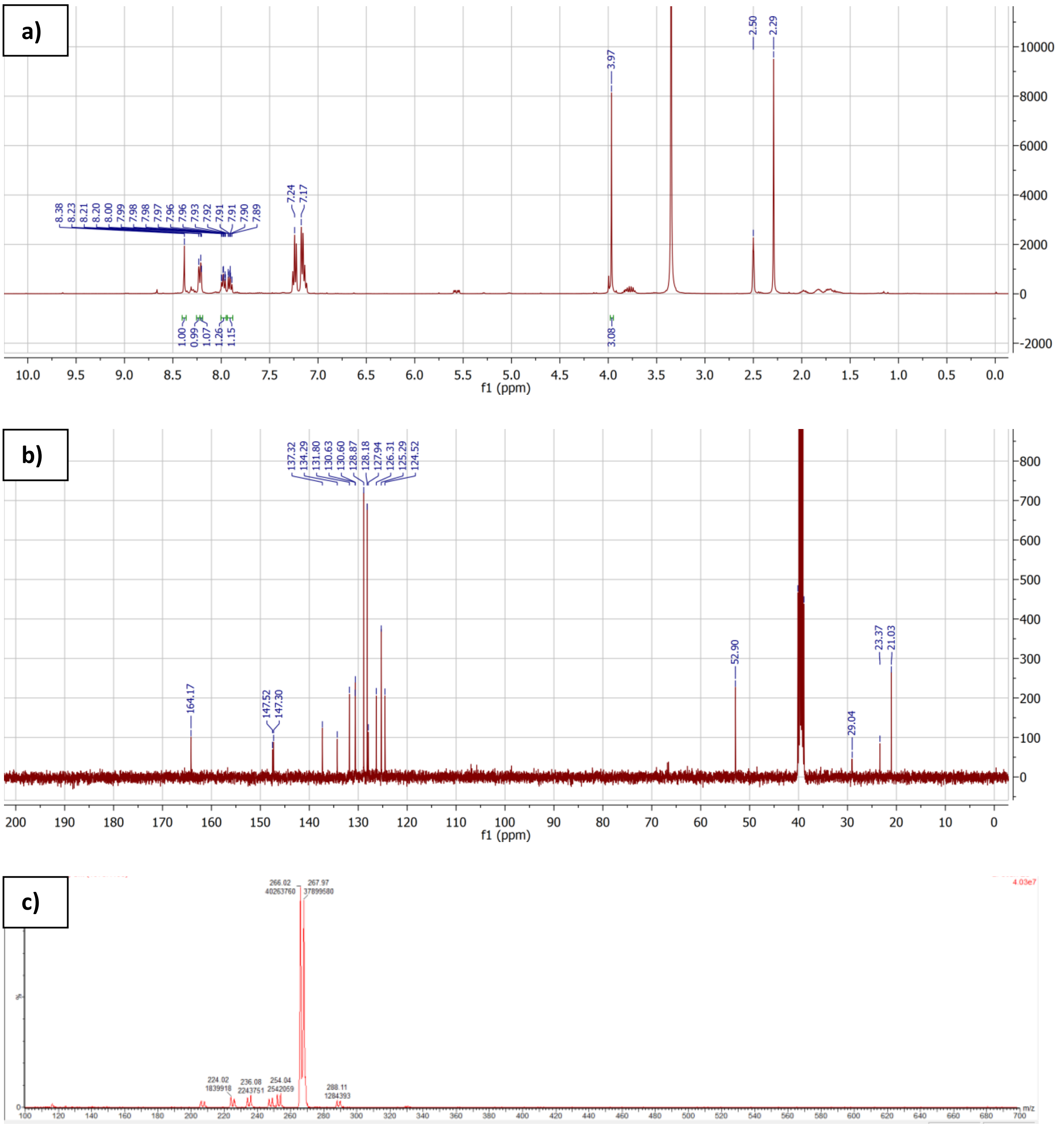

Figure S3: a) ^{1}H-NMR, b) ^{13}C-NMR and c) UPLC-MS spectrum of compound **6**.

Compound 7:

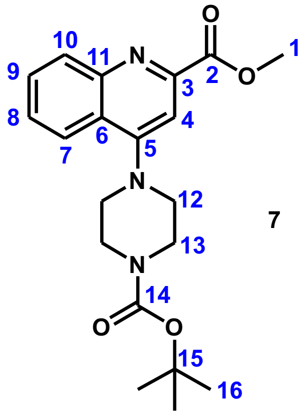

7

Under an N_2 atmosphere, methyl 4-bromoquinoline-2-carboxylate (**6**, 1.00 g, 3.76 mmol, 1.0 eq.), *tert*-butyl piperazine-1-carboxylate (707 mg, 3.76 mmol, 1.0 eq.), $Pd_2(dba)_3$ (196 mg, 0.23 mmol, 0.06 eq.), (*rac*)-BINAP (135 mg, 0.23 mmol, 0.06 eq.) and $CsCO_3$ (2.74 g, 8.41 mmol, 2.2 eq.) were mixed in dry 1,4-dioxane (30 ml) and heated under reflux for 15 h. The red mixture was filtered and the clear solution was evaporated to dryness. The resulting red oil was taken up in ethyl acetate and purified by flash column chromatography (SiO_2, ethyl acetate/cyclohexane 1:2 → 1:1). The fractions were concentrated and dried under reduced pressure to yield the product as a yellow-orange solid (**7**, 409 mg, 1.10 mmol, 29% yield).

^{1}H NMR (400 MHz, $CDCl_3$ δ/ppm): 8.26 (ddd, *J* = 8.5, 1.3, 0.6 Hz, 1H, **10**), 8.04 (ddd, *J* = 8.4, 1.5, 0.6 Hz, 1H, **7**), 7.73 (ddd, *J* = 8.5, 6.8, 1.4 Hz, 1H, **9**), 7.67 (s, 1H, **4**), 7.59 (ddd, *J* = 8.2, 6.8, 1.3 Hz, 1H, **8**), 4.06 (s, 3H, **1**), 3.78 – 3.69 (m, 4H, **13**), 3.30 – 3.19 (m, 4H, **12**), 1.50 (s, 9H, **16**). Solvents: Chloroform (7.26), DCM (5.29).

^{13}C NMR (101 MHz, $CDCl_3$ δ/ppm): 166.49 (1C, **2**), 158.01 (1C, **5**), 154.86 (1C, **14**), 149.14 (1C, **11**), 148.59 (1C, **3**), 131.60 (1C, **10**), 130.04 (1C, **9**), 127.61 (1C, **8**), 124.47 (1C, **6**), 123.49 (1C, **7**), 109.14 (1C, **4**), 80.37 (1C, **15**), 53.38 (1C, **1**), 52.26 (2C, **12**), 43.63 (2C, **13**, extrapolated from HMQC spectrum), 28.57 (3C, **16**). Solvents: Chloroform (77.48, 77.16, 76.84).

HRMS (ESI-MS, pos.) m/z: $[M+H]^+$ calculated for $C_{20}H_{26}N_3O_4$: 372.1918, found: 372.1923.

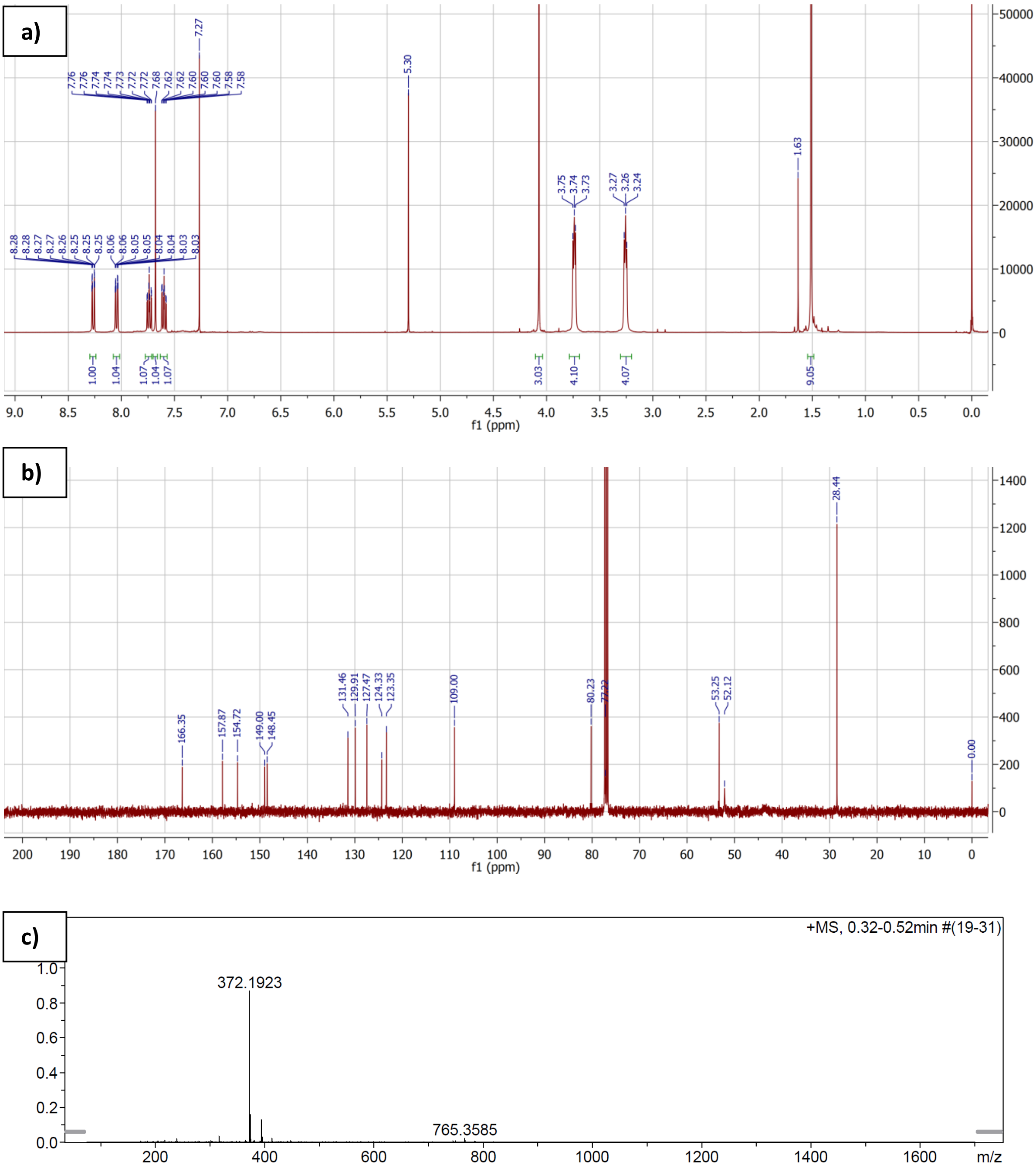

Figure S4: a) ^{1}H-NMR, b) ^{13}C-NMR and c) HRMS spectrum of compound **7**.

Compound 8:

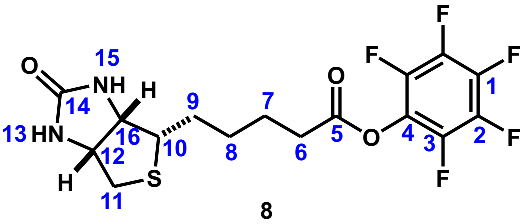

8

To a dispersion of D-biotin (1.00 g, 4.08 mmol, 1.0 eq.) in DMF (25 ml), triethylamine (1.00 ml, 0.73 g, 7.17 mmol, 1.8 eq.) was added at 0°C. Pentafluorophenyl trifluoroacetate (1.00 ml, 1.63 g, 5.81 mmol, 1.4 eq.) was slowly added, which led to the formation of a pink solution. The reaction mixture was allowed to warm to room temperature and was further stirred for 2 h, whereupon a white precipitate formed. Diethyl ether (80 ml) was added, the precipitate was filtered, washed with diethyl ether (80 ml) and dried under reduced pressure to yield the product as a white solid (**8**, 998 mg, 2.43 mmol, 60% yield).

^{1}H NMR (400 MHz, DMSO-*d*$_6$ δ/ppm): 6.45 (s, 1H, **15**), 6.37 (s, 1H, **13**), 4.36 – 4.27 (m, 1H, **12**), 4.19 – 4.11 (m, 1H, **16**), 3.17 – 3.08 (m, 1H, **10**), 2.87 – 2.81 (m, 1H, **11**), 2.79 (t, *J* = 7.6 Hz, 2H, **6**), 2.58 (d, *J* = 12.4 Hz, 1H, **11**), 1.78 – 1.34 (m, 6H, **7 + 8 + 9**). Solvents: Water (3.32), DMSO (2.50).

^{13}C NMR (101 MHz, DMSO-*d*$_6$ δ/ppm): 169.51 (1C, **5**), 162.67 (1C, **14**), 61.02 (1C, **16**), 59.17 (1C, **12**), 55.25 (1C, **10**), 39.78 (1C, **11**, extrapolated from HMQC spectrum), 32.30 (1C, **6**), 27.92 (1C, **9**), 27.68 (1C, **8**), 24.30 (1C, **7**). Solvents: DMSO (40.15, 39.94, 39.73, 39.52, 39.31, 39.10, 38.89). The signals for the quaternary carbons **1** - **4** could not be resolved.

^{19}**F NMR** (376 MHz, DMSO-d_6 δ/ppm): -153.59 (d, *J* = 19.1 Hz, 2F, **3**), -158.12 (t, *J* = 23.1 Hz, 1F, **1**), -162.63 (dd, *J* = 23.3, 19.1 Hz, 2F, **2**).

HRMS (ESI-MS, pos.) m/z: $[M+Na]^+$ calculated for $C_{16}H_{15}N_2O_3SF_5Na$: 433.0616, found: 433.0616.

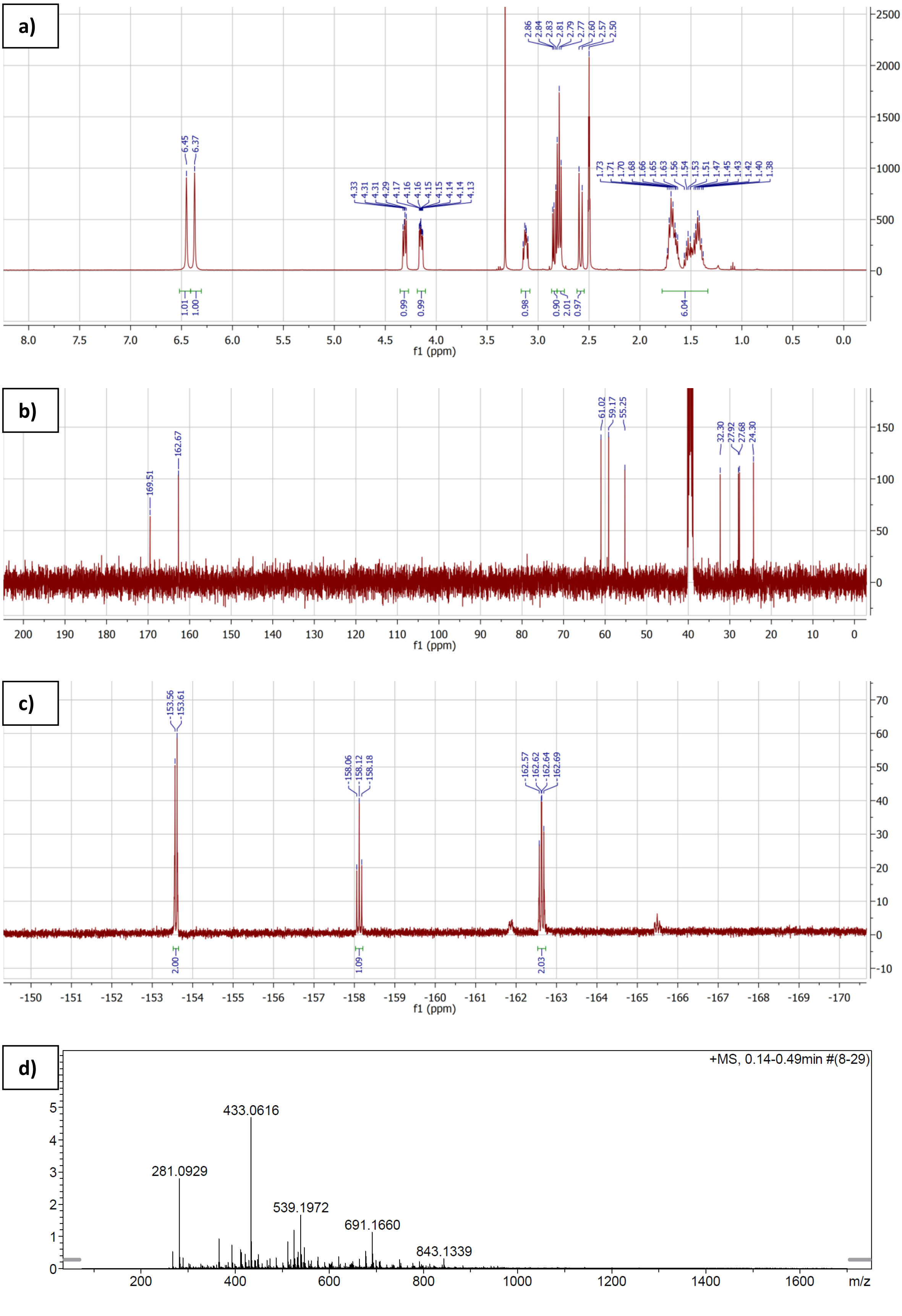

Figure S5: a) ^{1}H-NMR, b) ^{13}C-NMR, c) ^{19}F-NMR and d) HRMS spectrum of compound **8**.

Compound 9:

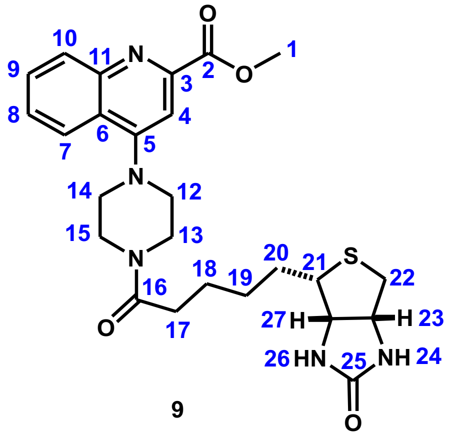

9

Methyl 4-(4-(tert-butoxycarbonyl)piperazin-1-yl)quinoline-2-carboxylate (**7**, 0.40 g, 1.09 mmol, 1.0 eq.) and triisopropylsilane (0.44 ml, 0.35 g, 2.18 mmol, 2.0 eq., scavenger for carbocations) were dissolved in DCM (2 ml) and treated with concentrated trifluoroacetic acid (2 ml). The red solution was stirred for 1 h at room temperature and evaporated to dryness. The orange oil was dissolved in DCM (2 ml). Addition of diethyl ether (20 ml) led to the formation of a yellow precipitate which was filtered, washed with diethyl ether (2 x 10 ml) and dried under reduced pressure to obtain an off-white solid (0.52 g). This solid was dissolved in DMF (5 ml), followed by the addition of *N*,*N*-diisopropylethylamine (0.95 ml, 0.70 g, 5.44 mmol, 5.0 eq.) and D-biotin pentafluorophenyl ester (**8**, 0.44 g, 1.09 mmol, 1.0 eq.). The reaction mixture was stirred for 24 h at room temperature (until no more D-biotin pentafluorophenyl ester **8** was detectable on TLC (DCM/MeOH 10:1)) and evaporated to dryness to afford a brown oil. Addition of diethyl ether (20 ml) led to the precipitation of an off-white solid, which was filtered and washed with diethyl ether (4 x 50 ml). The solid was then dissolved in DCM (20 ml), washed with saturated $NaHCO_3$ (20 ml) and water (20 ml).

The organic fraction was dried over Na_2SO_4. The solvent was evaporated under reduced pressure to yield the product as a pale yellow solid (**9**, 224 mg, 0.85 mmol, 78% yield).

^{1}H NMR (400 MHz, Methanol-*d*$_4$ δ/ppm): 8.18 (dd, *J* = 5.6, 0.8 Hz, 1H, **7**), 8.16 (dd, *J* = 5.4, 0.6 Hz, 1H, **10**), 7.79 (ddd, *J* = 8.5, 6.9, 1.4 Hz, 1H, **9**), 7.68 (ddd, *J* = 8.2, 6.8, 1.2 Hz, 1H, **8**), 7.67 (s, 1H, **4**), 4.50 (ddd, *J* = 7.9, 5.0, 1.0 Hz, 1H, **23**), 4.32 (dd, *J* = 7.9, 4.4 Hz, 1H, **27**), 4.03 (s, 3H, **1**), 3.92 (t, *J* = 4.8 Hz, 2H, **13 or 15**), 3.88 (t, *J* = 5.0 Hz, 2H, **13 or 15**), 3.39 – 3.33 (m, 2H, **12 or 14**), 3.30 – 3.27 (m, 2H, **12 or 14**), 3.23 (ddd, *J* = 8.8, 5.9, 4.4 Hz, 1H, **21**), 2.93 (dd, *J* = 12.8, 5.0 Hz, 1H, **22**), 2.71 (d, *J* = 12.6 Hz, 1H, **22**), 2.51 (t, *J* = 7.4 Hz, 2H, **17**), 1.83 – 1.59 (m, 4H, **18 + 20**), 1.56 – 1.45 (m, 2H, **19**). Solvents: DCM (5.49), Water (4.86), DMF (7.97, 2.99, 2.86).

^{13}C NMR (101 MHz, Methanol-*d*$_4$ δ/ppm): 174.24 (1C, **16**), 167.04 (1C, **2**), 166.14 (1C, **25**), 159.64 (1C, **5**), 149.97 (1C, **11**), 149.48 (1C, **3**), 131.56 (1C, **9**), 131.15 (1C, **10**), 128.86 (1C, **8**), 125.46 (1C, **6**), 125.03 (1C, **7**), 109.78 (1C, **4**), 63.41 (1C, **27**), 61.68 (1C, **23**), 57.08 (1C, **21**), 53.48 (1C, **1**), 53.41 (1C, **12 or 14**), 53.15 (1C, **12 or 14**), 46.88 (1C, **13 or 15**), 42.83 (1C, **13 or 15**), 41.09 (1C, **22**), 33.75 (1C, **17**), 29.94 (1C, **19**), 29.60 (1C, **20**), 26.41 (1C, **18**). Solvents: Methanol (49.68, 49.46, 49.25, 49.04, 48.82, 48.61, 48.40).

HRMS (ESI-MS, pos.) m/z: $[M+H]^+$ calculated for $C_{25}H_{32}N_5O_4S$: 498.2170, found: 498.2178.

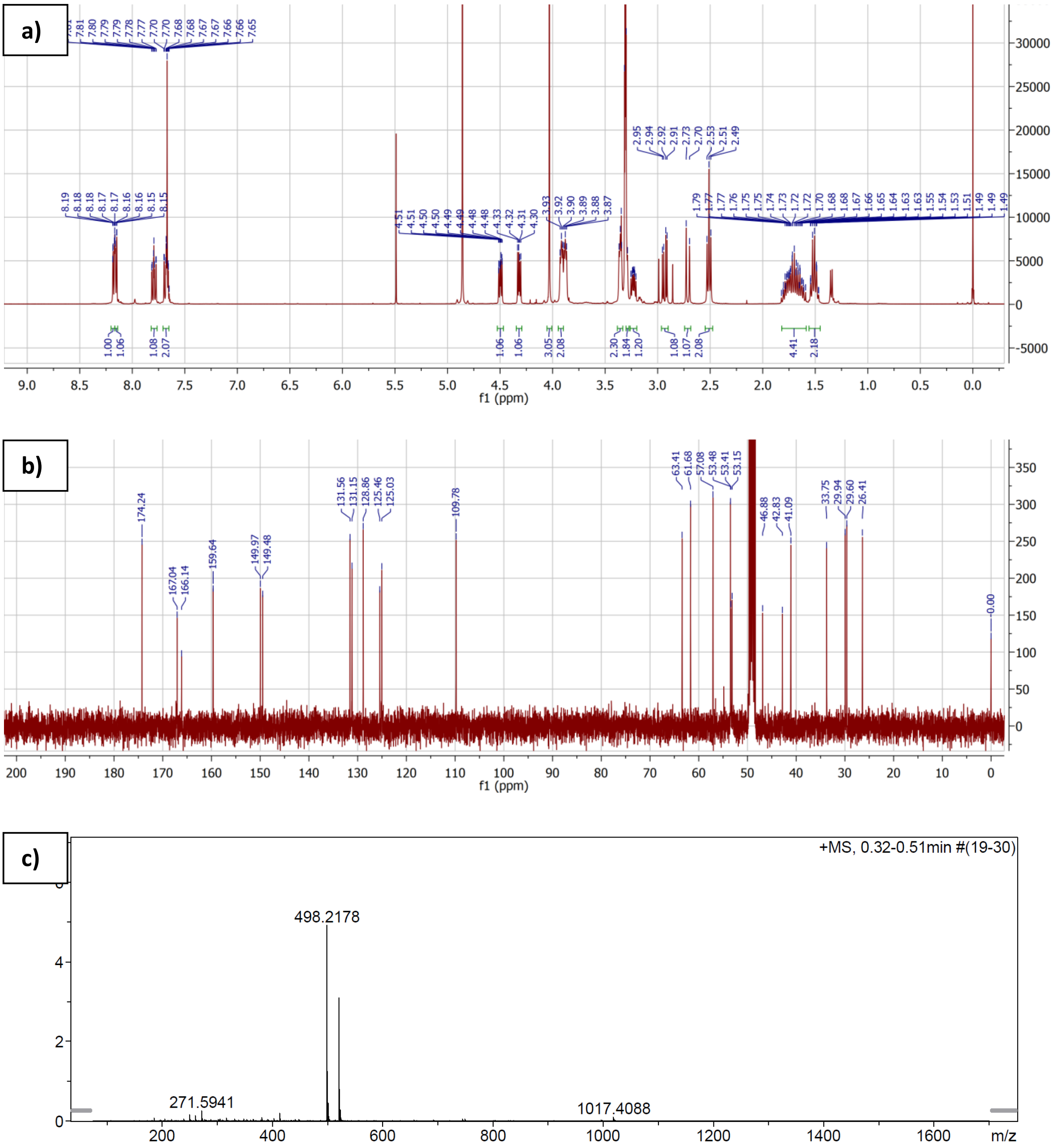

Figure S6: a) ^{1}H-NMR, b) ^{13}C-NMR and c) HRMS spectrum of compound **9**.

Compound 10:

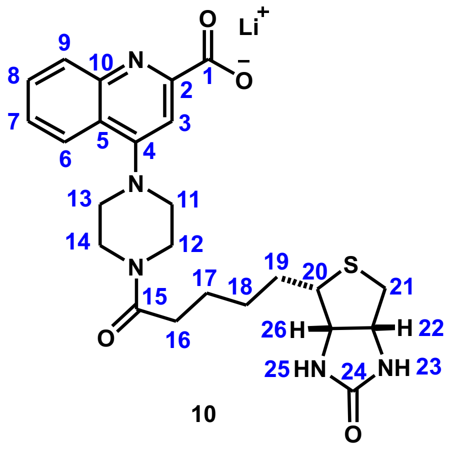

10

Methyl ester **9** (100 mg, 0.20 mmol, 1.0 eq.) was dissolved in MeOH (2 ml) and treated with LiOH·H_2O (16 mg, 0.40 mmol, 2.0 eq.). The reaction mixture was stirred for 22 h at room temperature (until no more starting material was visible on TLC (DCM/MeOH 10:1)). The mixture was filtered to remove the excess of insoluble LiOH. Addition of diethyl ether (5 ml) led to the formation of an off-white precipitate, which was washed with diethyl ether (3 x 5 ml) and dried under reduced pressure to obtain the product as an off-white solid (**10**, 80.9 mg, 0.17 mmol, 83% yield).

^{1}H NMR (400 MHz, Methanol-d_4 δ/ppm): 8.14 (dd, *J* = 8.4, 0.8 Hz, 1H, **6**), 8.07 (dd, *J* = 8.6, 0.9 Hz, 1H, **9**), 7.72 (ddd, *J* = 8.4, 6.8, 1.4 Hz, 1H, **8**), 7.66 (s, 1H, **3**), 7.59 (ddd, *J* = 8.3, 6.8, 1.2 Hz, 1H, **7**), 4.50 (ddd, *J* = 7.9, 5.0, 0.9 Hz, 1H, **22**), 4.32 (dd, *J* = 7.9, 4.4 Hz, 1H, **26**), 3.91 (t, *J* = 5.4 Hz, 2H, **12 or 14**), 3.88 (t, *J* = 4.7 Hz, 2H, **12 or 14**), 3.39 – 3.32 (m, 2H, **11 or 13**), 3.30 – 3.23 (m, 2H, **11 or 13**), 3.27 – 3.18 (m, 1H, **20**), 2.93 (dd, *J* = 12.7, 5.0 Hz, 1H, **21**), 2.71 (d, *J* = 12.7 Hz, 1H, **21**), 2.52 (t, *J* = 7.4 Hz, 2H, **16**), 1.84 – 1.58 (m, 4H, **17 + 19**), 1.57 – 1.45 (m, 2H, **18**). Solvents: Water (4.87), diethyl ether (3.49 + 1.17).

^{13}C NMR (101 MHz, Methanol-d_4 δ/ppm): 174.24 (1C, **15**), 173.04 (1C, **1**), 166.15 (1C, **24**), 159.12 (1C, **4**), 157.23 (1C, **2**), 149.54 (1C, **10**), 130.78 (1C, **8**), 130.66 (1C, **9**), 127.53 (1C, **7**), 124.80 (1C, **5 or 6**), 124.79 (1C, **5 or 6**), 109.87 (1C, **3**), 63.41 (1C, **26**), 61.69 (1C, **22**), 57.05 (1C, **20**), 53.37 (1C, **11 or 13**), 53.35 (1C, **11 or 13**), 46.99 (1C, **12 or 14**), 42.93 (1C, **12 or 14**), 41.08 (1C, **21**), 33.76 (1C, **16**), 29.92 (1C, **18**), 29.58 (1C, **19**), 26.42 (1C, **17**). Solvents: Methanol (49.68, 49.46, 49.25, 49.04, 48.82, 48.61, 48.40).

HRMS (ESI-MS, pos.) m/z: $[M+H+Na]^+$ calculated for $C_{24}H_{29}N_5O_4SNa$: 506.1832, found: 506.1838.

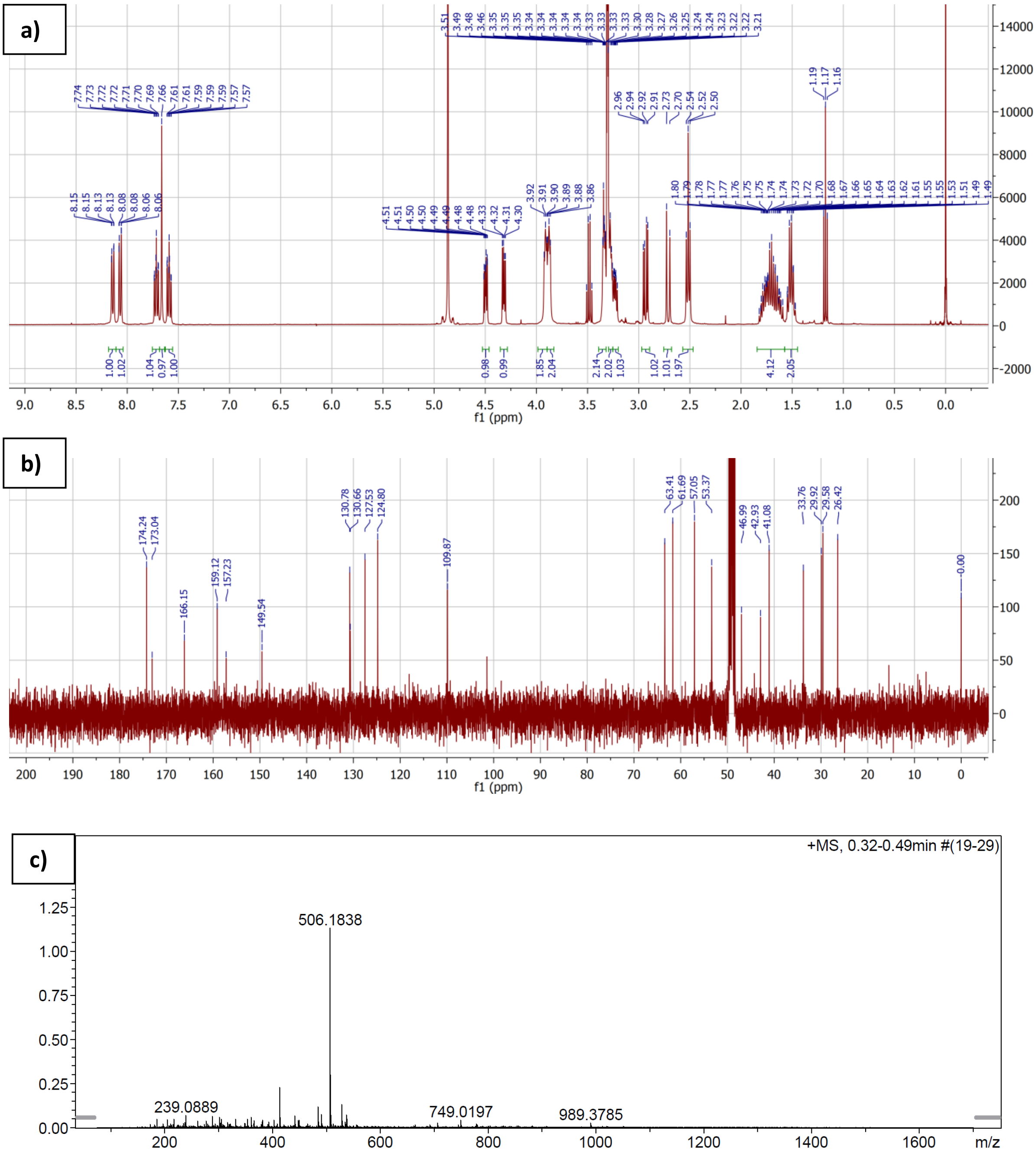

Figure S7: a) ^{1}H-NMR, b) ^{13}C-NMR and c) HRMS spectrum of compound **10**.

Compound 1:

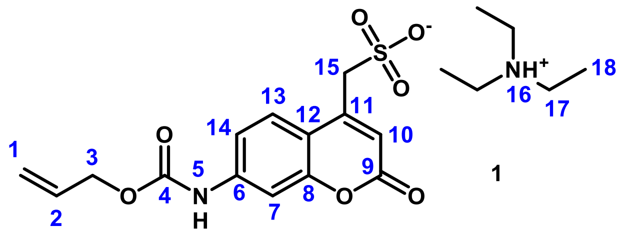

1

The synthesis of 7-aminocoumarin-4-methansulfonic acid **15** was performed as described by Kanaoka *et al.*[1] and Griffiths and Ryckelynck *et al.*[2] (Figure S8).

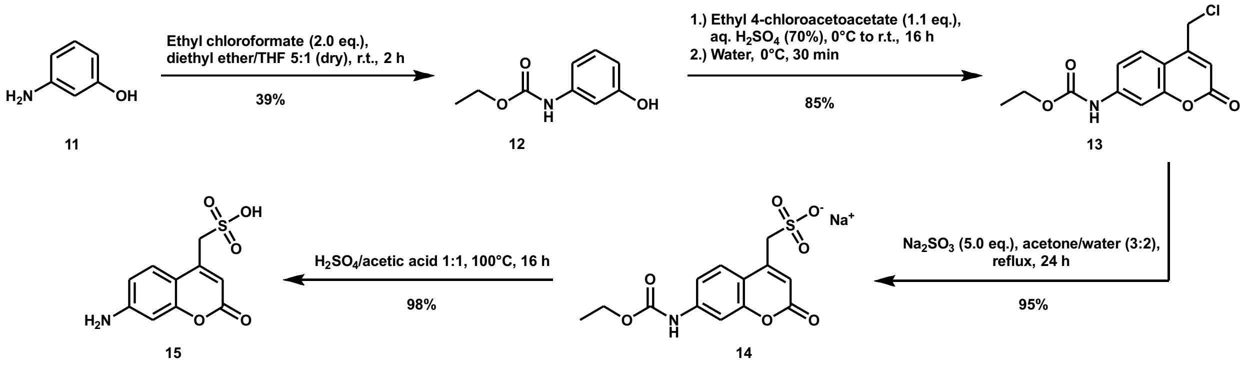

Figure S8: Synthesis of 7-aminocoumarin-4-methansulfonic acid **15**.

The synthesis of compound **1** was carried out as reported by Meggers *et al.*[3]

7-aminocoumarin-4-methansulfonic acid (**15**, 102 mg, 0.40 mmol, 1.0 eq.) was dispersed in triethylammonium bicarbonate buffer (1 M, pH 8.5, 4.0 ml, 4.0 mmol, 10.0 eq.). Allyl chloroformate (215 µl, 244 mg, 1.96 mmol, 5.0 eq.) was added dropwise at 0 °C. The mixture was stirred for 1.5 h at 0 °C and then for 2 h at room temperature. The obtained brownish solution was acidified with glacial acetic acid (0.5 ml) to pH 4 and then filtered. The filtrate was purified by preparative reverse phase HPLC (XSELECT™ CSH™ Prep C18 5 µm OBD™ 19 x 150 mm, water/acetonitrile containing 5 mM triethylamine and 5 mM acetic acid). The collected fractions were lyophilized to afford the product as a white solid (**1**, 46.8 mg, 0.11 mmol, 53% yield).

^{1}H NMR (400 MHz, DMSO-d_6 δ/ppm): 10.21 (s, 1H, **5**), 7.85 (d, *J* = 8.8 Hz, 1H, **13**), 7.54 (d, *J* = 2.1 Hz, 1H, **7**), 7.33 (dd, *J* = 8.8, 2.1 Hz, 1H, **14**), 6.24 (s, 1H, **10**), 6.00 (ddt, *J* = 17.2, 10.9, 5.5 Hz, 1H, **2**), 5.39 (dq, *J* = 17.2, 1.6 Hz, 1H, **1**), 5.26 (dq, *J* = 10.5, 1.3 Hz, 1H, **1**), 4.65 (dt, *J* = 5.5, 1.4 Hz, 2H, **3**), 3.99 (s, 2H, **15**), 3.08 (q, *J* = 7.3 Hz, 6H, **17**), 1.17 (t, *J* = 7.3 Hz, 9H, **18**). Solvents: Water (3.32), DMSO (2.50).

^{13}C NMR (101 MHz, DMSO-d_6 δ/ppm): 160.25 (1C, **9**), 154.08 (1C, **8**), 153.01 (1C, **4**), 149.99 (1C, **11**), 142.30 (1C, **6**), 132.97 (1C, **2**), 127.74 (1C, **13**), 117.94 (1C, **1**), 113.96 (1C, **10 or 12 or 14**), 113.91 (1C, **10 or 12 or 14**), 113.78 (1C, **10 or 12 or 14**), 104.19 (1C, **7**), 65.09 (1C, **3**), 53.14 (1C, **15**), 45.71 (3C, **17**), 8.62 (3C, **18**). Solvents: DMSO (40.15, 39.94, 39.73, 39.52, 39.31, 39.10, 38.89).

HRMS (ESI-MS, neg.) *m/z*: $[M-HNEt_3]^-$ calculated for $C_{14}H_{12}NO_7S$: 338.0340, found: 338.0342.

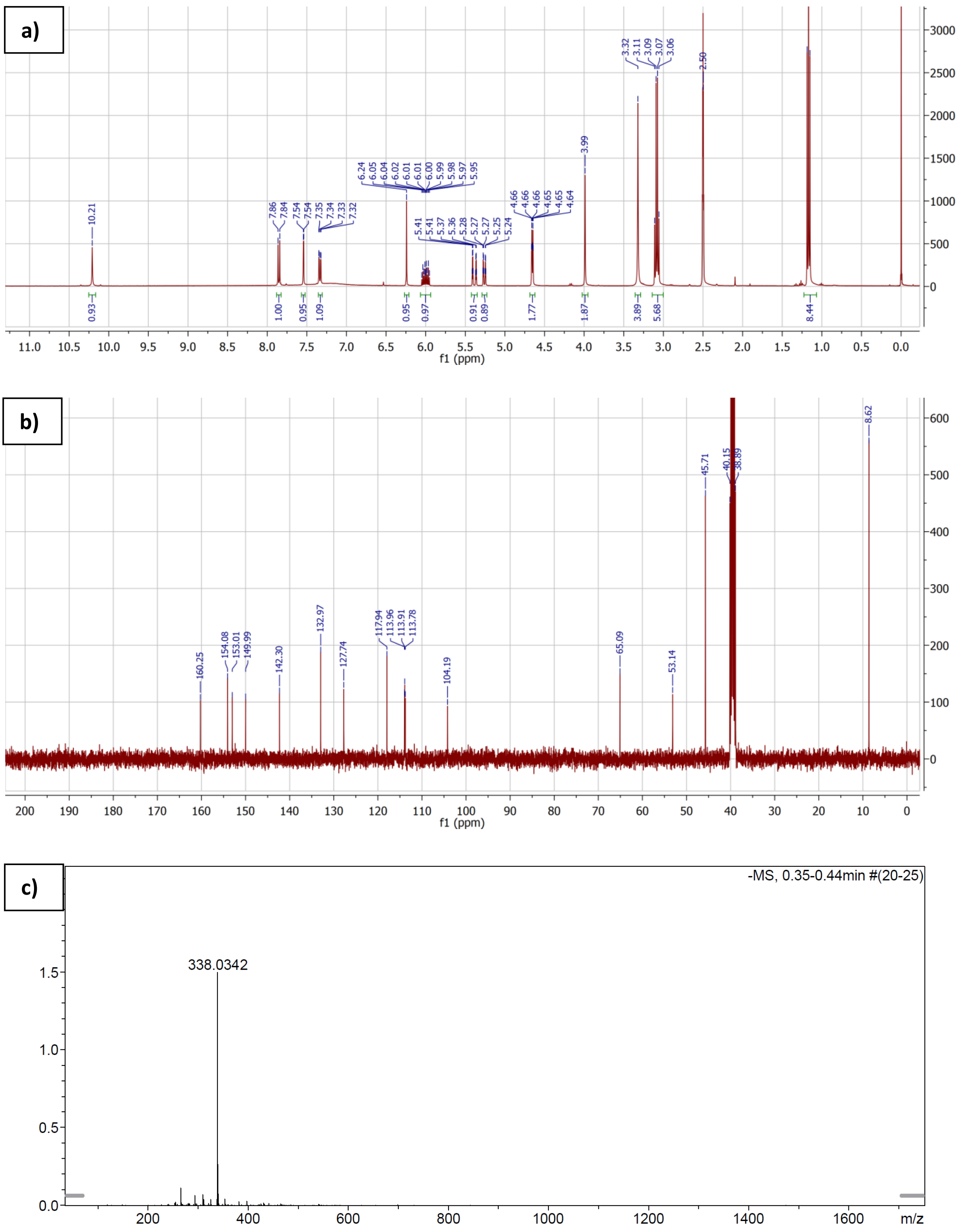

Figure S9: a) ^{1}H-NMR, b) ^{13}C-NMR and c) HRMS spectrum of compound **1**.

Compound 2:

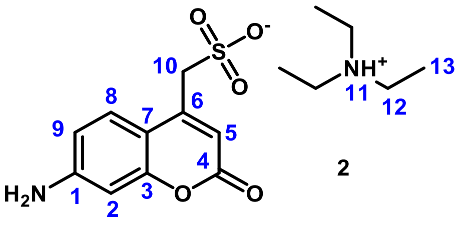

2

The synthesis of compound **2** was performed as described by Meggers *et al.*[3]

7-aminocoumarin-4-methansulfonic acid (**15**, 100 mg, 0.40 mmol, 1.0 eq.) was dispersed in triethylammonium bicarbonate buffer (1 M, pH 8.5, 4.0 ml, 4.0 mmol, 10.0 eq.). The resulting yellowish solution was acidified with glacial acetic acid (0.5 ml) to pH 4 and filtered. The filtrate was purified by preparative reverse phase HPLC (XSELECT™ CSH™ Prep C18 5 µm OBD™ 19 x 150 mm, water/acetonitrile containing 5 mM triethylamine and 5 mM acetic acid). The collected fractions were then lyophilized to obtain the product as a light yellow solid (**2**, 39.0 mg, 0.11 mmol, 28% yield).

^{1}H NMR (500 MHz, DMSO-d_6 δ/ppm): 8.86 (s, 1H, **11**), 7.55 (d, *J* = 8.7 Hz, 1H, **8**), 6.51 (dd, *J* = 8.7, 2.2 Hz, 1H, **9**), 6.38 (d, *J* = 2.2 Hz, 1H, **2**), 6.04 (s, 2H, **NH_2**), 5.92 (s, 1H, **5**), 3.86 (s, 2H, **10**), 3.08 (q, *J* = 7.2 Hz, 6H, **12**), 1.17 (t, *J* = 7.3 Hz, 9H, **13**). Solvents: Water (3.32), DMSO (2.50).

^{13}C NMR (126 MHz, DMSO-d_6 δ/ppm): 160.95 (1C, **4**), 155.75 (1C, **1 or 3**), 152.66 (1C, **1 or 3**), 150.51 (1C, **6**), 127.88 (1C, **8**), 110.84 (1C, **9**), 109.24 (1C, **5**), 108.60 (1C, **7**), 98.26 (1C, **2**), 53.32 (1C, **10**), 45.75 (1C, **12**), 8.66 (1C, **13**). Solvents: DMSO (40.02, 39.85, 39.69, 39.52, 39.35, 39.19, 39.02).

HRMS (ESI-MS, neg.) *m/z*: [M]$^-$ calculated for $C_{10}H_8NO_5S$: 254.0129, found: 254.0132.

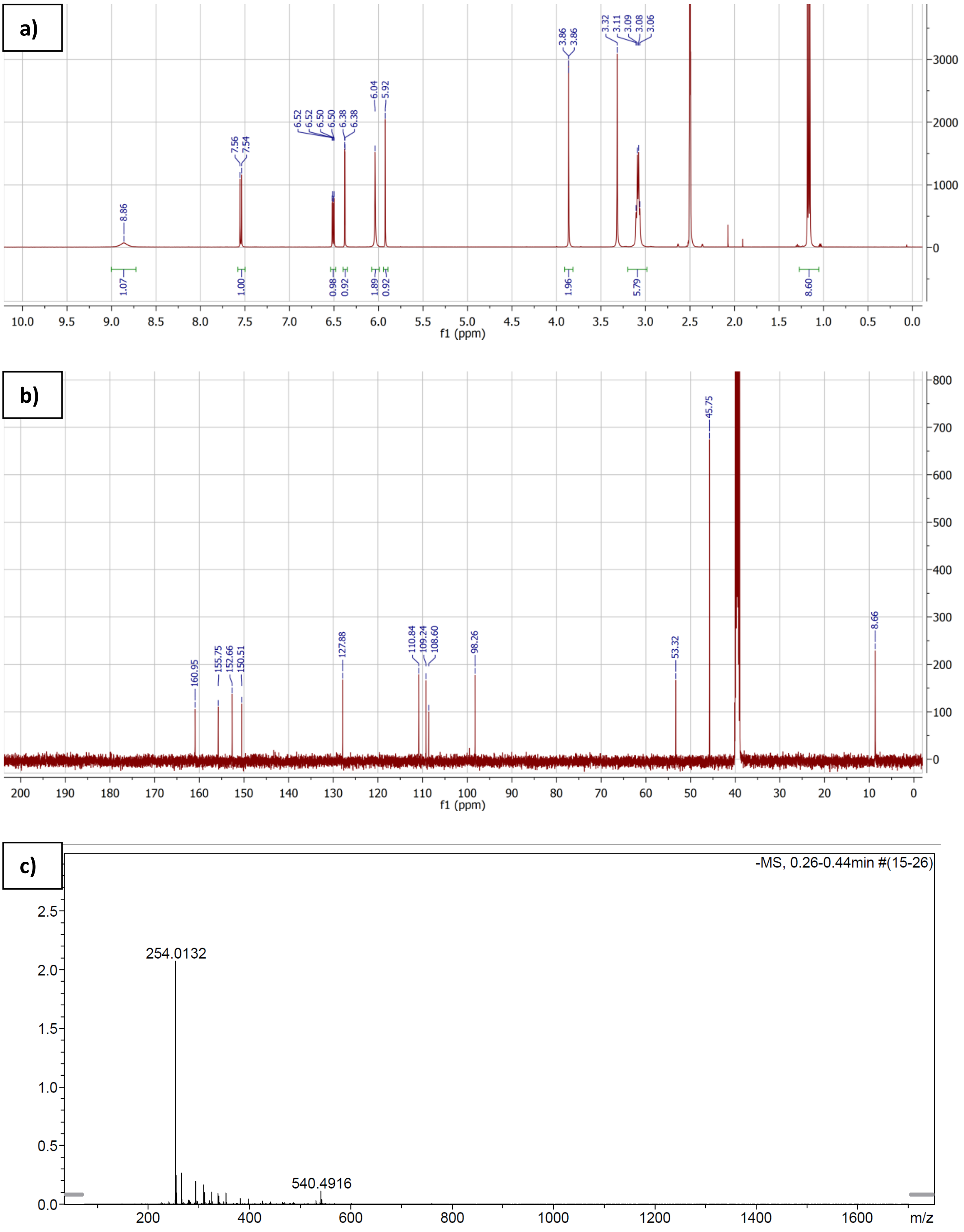

Figure S10: a) ^{1}H-NMR, b) ^{13}C-NMR and c) HRMS spectrum of compound **2**.

3. *In vitro* deprotection of substrate **1** using ADAse

Stock solutions:

- PBS buffer: 50 mM sodium phosphate, 0.9% NaCl, pH 7.4
- Ligand **10** (2 mM in DMF)
- Catalyst precursor $[CpRu(CH_3CN)_3]PF_6$ (2 mM in DMF)
- Cofactor **3** (200 µM in DMF): 20 uL ligand **10**, 20 µL catalyst precursor stock solution and 160 µL DMF were mixed and incubated for 10 min at room temperature.
- Streptavidin isoform (200 µM free biotin binding sites in PBS buffer)
- Substrate **1** (10 mM in water)
- Product **2** (10 mM in water)

In a 96-deep well plate, 233.75 µL PBS buffer, 2.5 µL streptavidin solution (2 µM final concentration of free biotin binding sites; 0.4 mol%) and 1.25 µL cofactor solution (1 µM final concentration; 0.2 mol% catalyst loading) were mixed and incubated at 28 °C for 5 min. Subsequently, 12.5 µL substrate solution (500 µM final concentration) were added and the reaction was incubated at 28 °C and 400 rpm. After various time points (1.3, 4.0, 7.0, 9.5, 24, 30 h) 3 µL of the reaction mixture were mixed with 147 µL PBS buffer in a black 96-well plate. The fluorescence was determined at λ_{ex} = 395nm and λ_{em} = 460nm. All measurements were carried out in triplicate (Figure 3b, Figure S11 and Figure S13b).

The conversion was determined by fluorescence using a calibration curve (Figure S12). The calibration curve was prepared by i) diluting substrate **1** and product **2** stock solutions to 10 µM each and ii) preparing aqueous solutions with varying ratios of substrate and product.

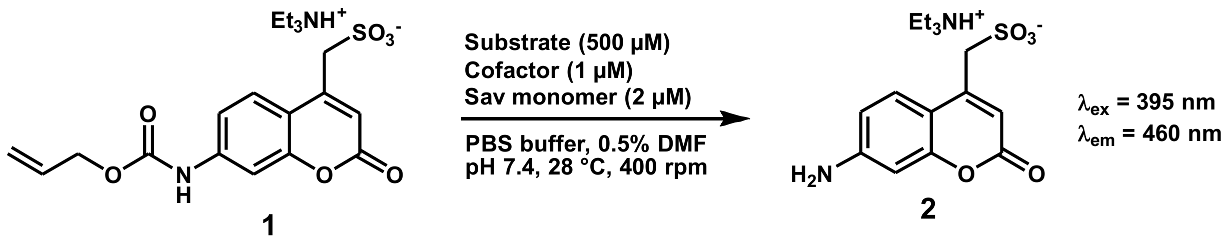

Figure S11: Optimized reaction conditions for *in vitro* uncaging of allylcoumarin **1** with ADAse.

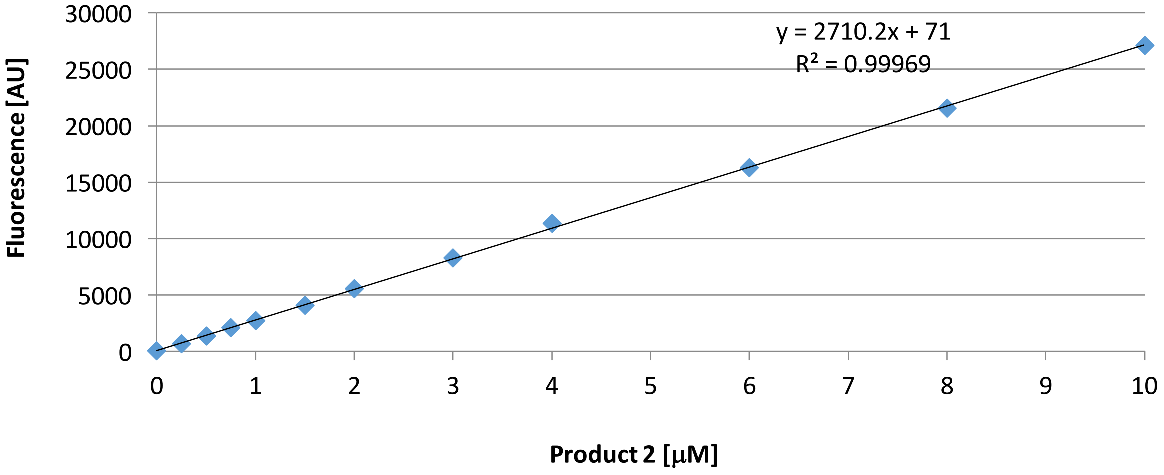

Figure S12: Calibration curve for the quantification of product **2** by fluorescence using the *in vitro* ADAse.

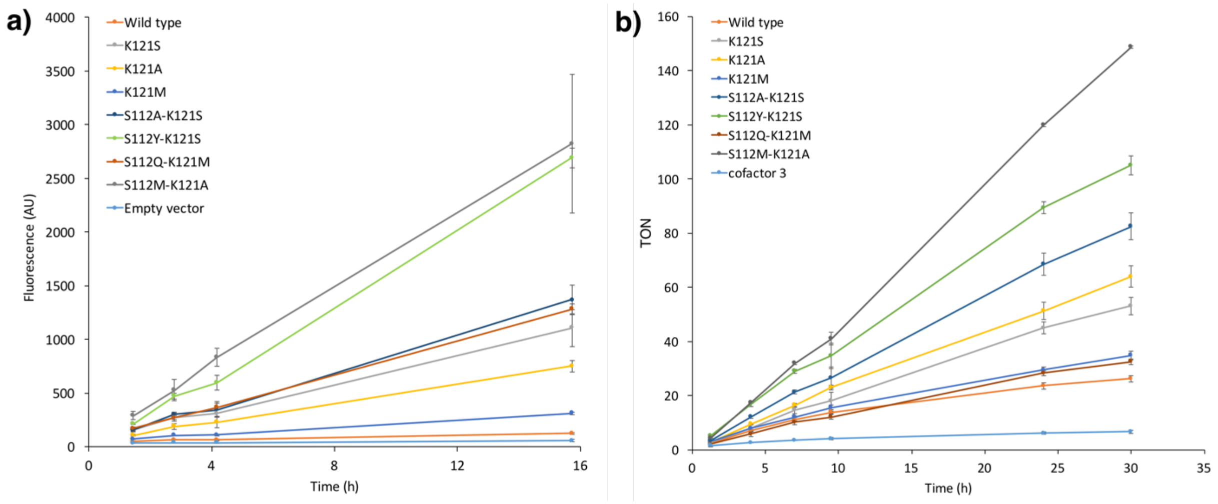

Figure S13: Time-course experiments for the uncaging of substrate **1** on cell surface (**a**) and with purified enzyme (**b**) with best hits from the activity screen of libraries K121**X** (K121S, K121A, K121M), S112**X**-K121S (S112A-K121S, S112Y-K121S), S112**X**-K121M (S112Q-K121M) and S112X-K121A (S112M-K121A). Catalysis experiments on the cell surface were carried out as described in section 10. The fluorescence was determined at varying time points (1.5, 2.8, 4.2, 16 h). All experiments were performed in triplicate. See text for experimental details. The TONs were determined using the calibration curve presented in Figure S12.

4. Cloning vector pBAD33_Lpp-OmpA-Sav for streptavidin display on *E. coli* surface

Cloning of target plasmid **pBAD33_Lpp-OmpA-Sav$_{\mathbf{WT(codon_optimized)}}$** (Figure S14 and Figure S15) was achieved using the following stepwise strategy:

Cloning Lpp-OmpA into plasmid pET30b_ompA-Sav$_{\mathbf{WT}}$

The Lpp-OmpA insert was amplified by PCR (Table S1 and Table S6) from the template plasmid pLpp-OmpA-SNAPtag (Table S2) using the primer pair Nde1-Lpp-OmpA and Nhe1-Lpp-OmpA (Table S7). The PCR product was purified using a PCR purification kit (Macherey-Nagel). The purified PCR product and plasmid pET30b_OmpA-Sav$_{WT}$[4] (Table S3) were digested with the restriction enzymes Nde1 and Nhe1 (purchased from NEB) following the manufacturer´s protocol (NEB). The digested insert and the backbone DNA was purified by agarose gel extraction and ligated using T4 DNA ligase following manufacturer´s protocol (NEB) to obtain plasmid pET30b_Lpp-OmpA-Sav$_{WT}$.

Cloning of Lpp-OmpA-Sav$_{\mathbf{WT}}$ into pBAD33_empty

Plasmids pET30b_Lpp-OmpA-Sav$_{WT}$ and pBAD33_empty (generously provided by the laboratory of Prof. Christoph Dehio, University of Basel) were digested using the restriction enzymes XbaI and HindIII following the manufacturer´s protocol (NEB). The digested DNA was purified by agarose gel extraction and ligated using T4 DNA ligase following the manufacturer´s protocol (NEB) to obtain the pBAD33_Lpp-OmpA-Sav$_{WT}$ plasmid.

Cloning of Sav $_{WT(codon\_optimized)}$ into pBAD33_Lpp-OmpA-Sav$_{WT}$

The plasmids pBAD33_Lpp-OmpA-Sav$_{WT}$ and pET30b_ompA-Sav$_{WT(codon_optimized)}$[5] were digested with the restriction enzymes NheI and HindIII following the manufacturer´s protocol (NEB). The digested DNA was purified by agarose gel extraction and ligated using T4 DNA ligase following the manufacturer´s protocol (NEB) to obtain plasmid pBAD33_Lpp-OmpA-Sav$_{WT(codon_optimized)}$ (Table S4).

Cloning Sav from pBAD33_Lpp-OmpA-Sav into pET11b_Sav for cytoplasmatic expression

The plasmid pET11b_empty[5] was digested with the restriction enzymes NheI and BamHI (NEB) and the linearized DNA backbone was purified from an agarose gel. The insert DNA was obtained from the plasmid pBAD33_Lpp-OmpA-Sav by digestion with the restriction enzymes NheI, BamHI and SphI followed by purification from an agarose gel of a band of size ~500 bp. Backbbone and insert DNA were ligated using T4 DNA ligase (NEB).

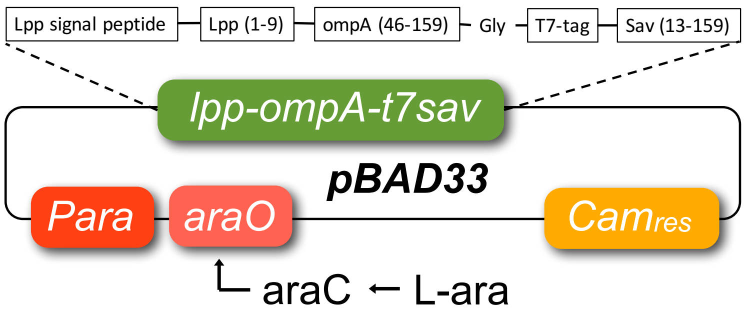

Figure S14: Map of the pBAD33 plasmid used for the expression of the Lpp-OmpA-T7-Sav surface construct on the outer membrane of *E. coli.* The plasmid pBAD33 contains the Para promoter, the araO operator, the lpp-ompA-T7-Sav gene cassette as well as a gene encoding resistance against the antibiotic chloramphenicol (Cam$_{res}$).

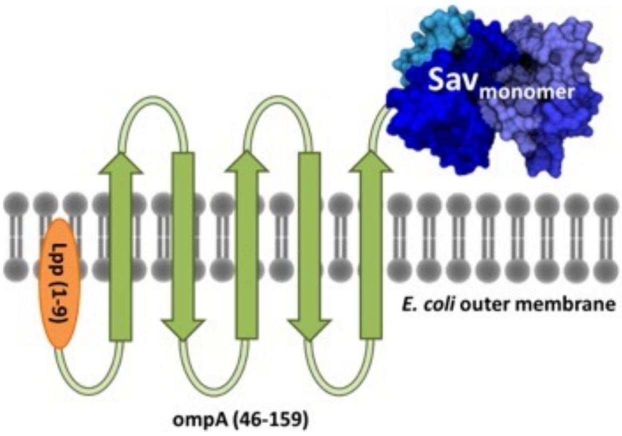

Figure S15: Schematic representation of the Lpp-OmpA-Sav system anchored in *E. coli*'s outer membrane.

Table S1: General PCR conditions for site-directed mutagenesis.

**Reagent**	**1 PCR reaction (µL)**
Q5 buffer, 5x	5
dNTPs (10 mM)	0.5
Forward primer	0.5
Reverse primer	0.5
Plasmid DNA (25 ng/µL)	1
Q5 Hot Start polymerase (NEB)	0.25
ddH2O	14.75
Q5 Enhancer, 5x	2.5
Total	25

Table S2: DNA sequence of the plasmid pLpp-OmpA-SNAPtag.

ctggccttttgctcacatgacccgacaccatcgaatggccagatgattaattcctaatttttgttgacactctatcattgatagagttatt
ttaccactccctatcagtgatagagaaaagtgaaatgaatagttcgacaaaaatctagaaaggagatatacacttcatgaaagcga
ccaaactggtgctgggcgcggtgattctgggcagcaccctgctggcgggctgcagcagcaacgcgaaaattgatcagaacaacaac
ggcccgacccatgaaaaccagctgggcgcgggcgcgtttggcggctatcaggtgaacccgtatgtgggctttgaaatgggctatgat
tggctgggccgcatgccgtataaaggcagcgtggaaaacggcgcgtataaagcgcagggcgtgcagctgaccgcgaaactgggct
atccgattaccgatgatctggatatttatacccgcctgggcggcatggtgtggcgcgcggataccaaaagcaacgtgtatggcaaaa
accatgataccggcgtgagcccggtgtttgcgggcggcgtggaatatgcgattaccccggaaattgcgaccggatcaatggacaaa
gattgcgaaatgaaacgtaccaccctggatagcccgctgggcaaactggaactgagcggctgcgaacagggcctgcatgaaatta
aactgctgggtaaaggcaccagcgcggccgatgcggttgaagttccggccccggccgccgtgctgggtggtccggaaccgctgatg
caggcgaccgcgtggctgaacgcgtattttcatcagccggaagcgattgaagaatttccggttccggcgctgcatcatccggtgtttc
agcaggagagctttacccgtcaggtgctgtggaaactgctgaaagtggttaaatttggcgaagtgattagctatcagcagctggcgg
ccctggcgggtaatccggcggccaccgccgccgttaaaaccgcgctgagcggtaacccggtgccgattctgattccgtgccatcgtgt
ggttagctctagcggtgcggttggcggttatgaaggtggtctggcggtgaaagagtggctgctggcccatgaaggtcatcgtctgggt
aaaccgggtctgggataagcttgacctgtgaagtgaaaaatggcgcacattgtgcgacatttttttgtctgccgtttaccgctactgc
gtcacggatctccacgcgccctgtagcggcgcattaagcgcggcgggtgtggtggttacgcgcagcgtgaccgctacacttgccagc
gccctagcgcccgctcctttcgctttcttcccttcctttctcgccacgttcgccggctttccccgtcaagctctaaatcgggggctcccttt
agggttccgatttagtgctttacggcacctcgaccccaaaaaacttgattagggtgatggttcacgtagtgggccatcgccctgatag
acggtttttcgccctttgacgttggagtccacgttctttaatagtggactcttgttccaaactggaacaacactcaaccctatctcggtct
attcttttgatttataagggattttgccgatttcggcctattggttaaaaaatgagctgatttaacaaaaatttaacgcgaattttaaca
aaatattaacgtttacaatttcaggtggcacttttcggggaaatgtgcgcggaacccctatttgtttatttttctaaatacattcaaatat
gtatccgctcatgagacaataaccctgataaatgcttcaataatattgaaaaaggaagagtatgagtattcaacatttccgtgtcgcc
cttattcccttttttgcggcattttgccttcctgtttttgctcacccagaaacgctggtgaaagtaaaagatgctgaagatcagttgggtg
cacgagtgggttacatcgaactggatctcaacagcggtaagatccttgagagttttcgccccgaagaacgttttccaatgatgagcac
ttttaaagttctgctatgtggcgcggtattatcccgtattgacgccgggcaagagcaactcggtcgccgcatacactattctcagaatg
acttggttgagtactcaccagtcacagaaaagcatcttacggatggcatgacagtaagagaattatgcagtgctgccataaccatga
gtgataacactgcggccaacttacttctgacaacgatcggaggaccgaaggagctaaccgcttttttgcacaacatgggggatcatg
taactcgccttgatcgttgggaaccggagctgaatgaagccataccaaacgacgagcgtgacaccacgatgcctgtagcaatggca
acaacgttgcgcaaactattaactggcgaactacttactctagcttcccggcaacaattgatagactggatggaggcggataaagtt
gcaggaccacttctgcgctcggcccttccggctggctggtttattgctgataaatctggagccggtgagcgtggctctcgcggtatcat
tgcagcactggggccagatggtaagccctcccgtatcgtagttatctacacgacggggagtcaggcaactatggatgaacgaaata
gacagatcgctgagataggtgcctcactgattaagcattggtaggaattaatgatgtctcgtttagataaaagtaaagtgattaacag

cgcattagagctgcttaatgaggtcggaatcgaaggtttaacaacccgtaaactcgcccagaagctaggtgtagagcagcctacatt
gtattggcatgtaaaaaataagcgggctttgctcgacgccttagccattgagatgttagataggcaccatactcacttttgccctttag
aaggggaaagctggcaagatttttacgtaataacgctaaaagttttagatgtgctttactaagtcatcgcgatggagcaaaagtac
atttaggtacacggcctacagaaaaacagtatgaaactctcgaaaatcaattagcctttttatgccaacaaggtttttcactagagaa
tgcattatatgcactcagcgcagtggggcattttactttaggttgcgtattggaagatcaagagcatcaagtcgctaaagaagaaag
ggaaacacctactactgatagtatgccgccattattacgacaagctatcgaattatttgatcaccaaggtgcagagccagccttctta
ttcggccttgaattgatcatatgcggattagaaaaacaacttaaatgtgaaagtgggtcttaaaagcagcataacctttttccgtgat
ggtaacttcactagtttaaaaggatctaggtgaagatcctttttgataatctcatgaccaaaatcccttaacgtgagttttcgttccact
gagcgtcagaccccgtagaaaagatcaaaggatcttcttgagatcctttttttctgcgcgtaatctgctgcttgcaaacaaaaaaacc
accgctaccagcggtggtttgtttgccggatcaagagctaccaactctttttccgaaggtaactggcttcagcagagcgcagatacca
aatactgtccttctagtgtagccgtagttaggccaccacttcaagaactctgtagcaccgcctacatacctcgctctgctaatcctgtta
ccagtggctgctgccagtggcgataagtcgtgtcttaccgggttggactcaagacgatagttaccggataaggcgcagcggtcgggc
tgaacggggggttcgtgcacacagcccagcttggagcgaacgacctacaccgaactgagatacctacagcgtgagctatgagaaa
gcgccacgcttcccgaagggagaaaggcggacaggtatccggtaagcggcagggtcggaacaggagagcgcacgagggagcttc
cagggggaaacgcctggtatctttatagtcctgtcgggtttcgccacctctgacttgagcgtcgatttttgtgatgctcgtcaggggg
gcggagcctatggaaaaacgccagcaacgcggcctttttacggttcctggccttttg

green: Lpp; blue: OmpA

Table S3: DNA sequence of the plasmid pET30b_ompA-Sav_{WT}.

tggcgaatgggacgcgccctgtagcggcgcattaagcgcggcgggtgtggtggttacgcgcagcgtgaccgctacacttgccagcg
ccctagcgcccgctcctttcgctttcttcccttcctttctcgccacgttcgccggctttccccgtcaagctctaaatcgggggctcccttta
gggttccgatttagtgctttacggcacctcgaccccaaaaaacttgattagggtgatggttcacgtagtgggccatcgccctgataga
cggtttttcgccctttgacgttggagtccacgttctttaatagtggactcttgttccaaactggaacaacactcaaccctatctcggtcta
ttcttttgatttataagggattttgccgatttcggcctattggttaaaaaatgagctgatttaacaaaaatttaacgcgaattttaacaa
aatattaacgtttacaatttcaggtggcacttttcggggaaatgtgcgcggaacccctatttgtttatttttctaaatacattcaaatatg
tatccgctcatgaattaattcttagaaaaactcatcgagcatcaaatgaaactgcaatttattcatatcaggattatcaataccatattt
ttgaaaaagccgtttctgtaatgaaggagaaaactcaccgaggcagttccataggatggcaagatcctggtatcggtctgcgattcc
gactcgtccaacatcaatacaacctattaatttcccctcgtcaaaaataaggttatcaagtgagaaatcaccatgagtgacgactga
atccggtgagaatggcaaaagtttatgcatttctttccagacttgttcaacaggccagccattacgctcgtcatcaaaatcactcgcat
caaccaaaccgttattcattcgtgattgcgcctgagcgagacgaaatacgcgatcgctgttaaaaggacaattacaaacaggaatc
gaatgcaaccggcgcaggaacactgccagcgcatcaacaatattttcacctgaatcaggatattcttctaatacctggaatgctgttt
tcccggggatcgcagtggtgagtaaccatgcatcatcaggagtacggataaaatgcttgatggtcggaagaggcataaattccgtca
gccagtttagtctgaccatctcatctgtaacatcattggcaacgctacctttgccatgtttcagaaacaactctggcgcatcgggcttcc
catacaatcgatagattgtcgcacctgattgcccgacattatcgcgagcccatttatacccatataaatcagcatccatgttggaattt
aatcgcggcctagagcaagacgtttcccgttgaatatggctcataacaccccttgtattactgtttatgtaagcagacagttttattgtt
catgaccaaaatcccttaacgtgagttttcgttccactgagcgtcagaccccgtagaaaagatcaaaggatcttcttgagatcctttttt
ttctgcgcgtaatctgctgcttgcaaacaaaaaaaccaccgctaccagcggtggtttgtttgccggatcaagagctaccaactctttttt
ccgaaggtaactggcttcagcagagcgcagataccaaatactgtccttctagtgtagccgtagttaggccaccacttcaagaactctg
tagcaccgcctacatacctcgctctgctaatcctgttaccagtggctgctgccagtggcgataagtcgtgtcttaccgggttggactca
agacgatagttaccggataaggcgcagcggtcgggctgaacggggggttcgtgcacacagcccagcttggagcgaacgacctaca
ccgaactgagatacctacagcgtgagctatgagaaagcgccacgcttcccgaagggagaaaggcggacaggtatccggtaagcgg
cagggtcggaacaggagagcgcacgagggagcttccagggggaaacgcctggtatctttatagtcctgtcgggtttcgccacctctg
acttgagcgtcgatttttgtgatgctcgtcaggggggcggagcctatggaaaaacgccagcaacgcggcctttttacggttcctggcc
ttttgctggccttttgctcacatgttctttcctgcgttatcccctgattctgtggataaccgtattaccgcctttgagtgagctgataccgc
tcgccgcagccgaacgaccgagcgcagcgagtcagtgagcgaggaagcggaagagcgcctgatgcggtattttctccttacgcatc
tgtgcggtatttcacaccgcatatatggtgcactctcagtacaatctgctctgatgccgcatagttaagccagtatacactccgctatc
gctacgtgactgggtcatggctgcgccccgacacccgccaacacccgctgacgcgccctgacgggcttgtctgctcccggcatccgct
tacagacaagctgtgaccgtctccgggagctgcatgtgtcagaggttttcaccgtcatcaccgaaacgcgcgaggcagctgcggtaa
agctcatcagcgtggtcgtgaagcgattcacagatgtctgcctgttcatccgcgtccagctcgttgagtttctccagaagcgttaatgt
ctggcttctgataaagcgggccatgttaagggcggttttttcctgtttggtcactgatgcctccgtgtaagggggatttctgttcatggg

ggtaatgataccgatgaaacgagagaggatgctcacgatacgggttactgatgatgaacatgcccggttactggaacgttgtgagg
gtaaacaactggcggtatggatgcggcgggaccagagaaaaatcactcagggtcaatgccagcgcttcgttaatacagatgtaggt
gttccacagggtagccagcagcatcctgcgatgcagatccggaacataatggtgcagggcgctgacttccgcgtttccagactttac
gaaacacggaaaccgaagaccattcatgttgttgctcaggtcgcagacgttttgcagcagcagtcgcttcacgttcgctcgcgtatcg
gtgattcattctgctaaccagtaaggcaaccccgccagcctagccgggtcctcaacgacaggagcacgatcatgcgcacccgtggg
gccgccatgccggcgataatggcctgcttctcgccgaaacgtttggtggcgggaccagtgacgaaggcttgagcgagggcgtgcaa
gattccgaataccgcaagcgacaggccgatcatcgtcgcgctccagcgaaagcggtcctcgccgaaaatgacccagagcgctgccg
gcacctgtcctacgagttgcatgataaagaagacagtcataagtgcggcgacgatagtcatgccccgcgcccaccggaaggagctg
actgggttgaaggctctcaagggcatcggtcgagatcccggtgcctaatgagtgagctaacttacattaattgcgttgcgctcactgc
ccgctttccagtcgggaaacctgtcgtgccagctgcattaatgaatcggccaacgcgcggggagaggcggtttgcgtattgggcgcc
agggtggtttttcttttcaccagtgagacgggcaacagctgattgcccttcaccgcctggccctgagagagttgcagcaagcggtcca
cgctggtttgccccagcaggcgaaaatcctgtttgatggtggttaacggcgggatataacatgagctgtcttcggtatcgtcgtatccc
actaccgagatgtccgcaccaacgcgcagcccggactcggtaatggcgcgcattgcgcccagcgccatctgatcgttggcaaccag
catcgcagtgggaacgatgccctcattcagcatttgcatggtttgttgaaaaccggacatggcactccagtcgccttcccgttccgcta
tcggctgaatttgattgcgagtgagatatttatgccagccagccagacgcagacgcgccgagacagaacttaatgggcccgctaac
agcgcgatttgctggtgacccaatgcgaccagatgctccacgcccagtcgcgtaccgtcttcatgggagaaaataatactgttgatg
ggtgtctggtcagagacatcaagaaataacgccggaacattagtgcaggcagcttccacagcaatggcatcctggtcatccagcgg
atagttaatgatcagcccactgacgcgttgcgcgagaagattgtgcaccgccgctttacaggcttcgacgccgcttcgttctaccatcg
acaccaccacgctggcacccagttgatcggcgcgagatttaatcgccgcgacaatttgcgacggcgcgtgcagggccagactggag
gtggcaacgccaatcagcaacgactgtttgcccgccagttgttgtgccacgcggttgggaatgtaattcagctccgccatcgccgctt
ccactttttcccgcgttttcgcagaaacgtggctggcctggttcaccacgcgggaaacggtctgataagagacaccggcatactctgc
gacatcgtataacgttactggtttcacattcaccaccctgaattgactctcttccgggcgctatcatgccataccgcgaaaggttttgcg
ccattcgatggtgtccgggatctcgacgctctcccttatgcgactcctgcattaggaagcagcccagtagtaggttgaggccgttgag
caccgccgccgcaaggaatggtgcatgcaaggagatggcgcccaacagtcccccggccacggggcctgccaccatacccacgccg
aaacaagcgctcatgagcccgaagtggcgagcccgatcttccccatcggtgatgtcggcgatataggcgccagcaaccgcacctgt
ggcgccggtgatgccggccacgatgcgtccggcgtagaggatcgagatcgatctcgatcccgcgaaattaatacgactcactatag
gggaattgtgagcggataacaattcccctctagaaataattttgtttaactttaagaaggagatatacatatgaaaaagacagctatc
gcgattgcagtggcactggctggtttcgctaccgtagcgcaggccgctagcatgactggtggacagcaaatgggtcgggatcaggcc
ggcatcaccggcacctggtacaaccagctcggctcgaccttcatcgtgaccgcgggcgccgacggcgccctgaccggaacctacga
gtcggccgtcggcaacgccgagagccgctacgtcctgaccggtcgttacgacagcgccccggccaccgacggcagcggcaccgccc
tcggttggacggtggcctggaagaataactaccgcaacgcccactccgcgaccacgtggagcggccagtacgtcggcggcgccga
ggcgaggatcaacacccagtggctgctgacctccggcaccaccgaggccaacgcctggaagtccacgctggtcggccacgacacct

tcaccaaggtgaagccgtccgccgcctccatcgacgcggcgaagaaggccggcgtcaacaacggcaacccgctcgacgccgttca

gcagtaataaggatccgaattcgagctccgtcgacaagcttgcggccgcactcgagcaccaccaccaccaccactgagatccggct

gctaacaaagcccgaaaggaagctgagttggctgctgccaccgctgagcaataactagcataaccccttggggcctctaaacgggt

cttgaggggttttttgctgaaaggaggaactatatccggat

pink: restriction enzyme digestion site (Nde1 and Nhe1); yellow: T7Sav

Table S4: DNA sequence of plasmid pBAD33_Lpp-OmpA-SavWT$_{\text{(codon optimized)}}$.

Natcgatgcataatgtgcctgtcaaatggacgaagcagggattctgcaaaccctatgctactccgtcaagccgtcaattgtctgattc
gttaccaattatgacaacttgacggctacatcattcactttttcttcacaaccggcacggaactcgctcgggctggccccggtgcatttt
ttaaatacccgcgagaaatagagttgatcgtcaaaaccaacattgcgaccgacggtggcgataggcatccgggtggtgctcaaaag
cagcttcgcctggctgatacgttggtcctcgcgccagcttaagacgctaatccctaactgctggcggaaaagatgtgacagacgcga
cggcgacaagcaaacatgctgtgcgacgctggcgatatcaaaattgctgtctgccaggtgatcgctgatgtactgacaagcctcgcg
tacccgattatccatcggtggatggagcgactcgttaatcgcttccatgcgccgcagtaacaattgctcaagcagatttatcgccagc
agctccgaatagcgcccttccccttgcccggcgttaatgatttgcccaaacaggtcgctgaaatgcggctggtgcgcttcatccgggc
gaaagaaccccgtattggcaaatattgacggccagttaagccattcatgccagtaggcgcgcggacgaaagtaaacccactggtga
taccattcgcgagcctccggatgacgaccgtagtgatgaatctctcctggcgggaacagcaaaatatcacccggtcggcaaacaaa
ttctcgtccctgatttttcaccaccccctgaccgcgaatggtgagattgagaatataacctttcattcccagcggtcggtcgataaaaa
aatcgagataaccgttggcctcaatcggcgttaaacccgccaccagatgggcattaaacgagtatcccggcagcaggggatcatttt
gcgcttcagccatacttttcatactcccgccattcagagaagaaaccaattgtccatattgcatcagacattgccgtcactgcgtctttt
actggctcttctcgctaaccaaaccggtaaccccgcttattaaaagcattctgtaacaaagcgggaccaaagccatgacaaaaacg
cgtaacaaaagtgtctataatcacggcagaaaagtccacattgattatttgcacggcgtcacactttgctatgccatagcatttttatc
cataagattagcggatcctacctgacgctttttatcgcaactctctactgtttctccatacccgtttttttgggctagcgaattcgagctc
ggtacccggggatcctctagaaaggagatatacacttcatgaaagcgaccaaactggtgctgggcgcggtgattctgggcagcacc
ctgctggcgggctgcagcagcaacgcgaaaattgatcagaacaacaacggcccgacccatgaaaaccagctgggcgcgggcgcgt
ttggcggctatcaggtgaacccgtatgtgggctttgaaatgggctatgattggctgggccgcatgccgtataaaggcagcgtggaaa
acggcgcgtataaagcgcagggcgtgcagctgaccgcgaaactgggctatccgattaccgatgatctggatatttatacccgcctgg
gcggcatggtgtggcgcgcggataccaaaagcaacgtgtatggcaaaaaccatgataccggcgtgagcccggtgtttgcgggcggc
gtggaatatgcgattaccccggaaattgcgaccggagctagcatgaccggtggccagcagatgggtcgtgatcaggcaggtattac
cggcacctggtataatcagctgggtagcacctttattgttaccgcaggcgcagatggtgcactgaccggtacgtatgaaagcgcagtt
ggtaatgcagaaagccgttatgttctgacaggtcgttatgatagcgcaccggcaaccgatggtagcggcaccgcactgggttggacc
gttgcatggaaaaataactatcgtaatgcacatagcgcaaccacctggtcaggtcagtatgttggtggtgcagaagcacgcattaat
acccagtggctgctgaccagcggcaccaccgaagcaaatgcctggaaaagcaccctggttggtcatgatacctttaccaaagttaa
accgagcgcagcaagcattgatgcagcaaaaaaagccggtgtgaataatggtaatccgctggatgcagttcagcagtaataagga
tccgaattcgagctccgtcgacaagcttggctgttttggcggatgagagaagattttcagcctgatacagattaaatcagaacgcag
aagcggtctgataaaacagaatttgcctggcggcagtagcgcggtggtcccacctgaccccatgccgaactcagaagtgaaacgcc
gtagcgccgatggtagtgtggggtctccccatgcgagagtagggaactgccaggcatcaaataaaacgaaaggctcagtcgaaag
actgggcctttcgttttatctgttgtttgtcggtgaacgctctcctgagtaggacaaatccgccgggagcggatttgaacgttgcgaag
caacggcccggagggtggcgggcaggacgcccgccataaactgccaggcatcaaattaagcagaaggccatcctgacggatggcc

tttttgcgtttctacaaactcttttgtttatttttctaaatacattcaaatatgtatccgctcatgagacaataaccctgataaatgcttca
ataatattgaaaaaggaagagtatgagtattcaacatttccgtgtcgcccttattcccttttttgcggcattttgccttcctgtttttgctc
acccagaaacgctggtgaaagtaaaagatgctgaagatcagttggggcaaactattaactggcgaactacttactctagcttcccgg
caacaattaatagactggatggaggcggataaagttgcaggaccacttctgcgctcggcccttccggctggctggtttattgctgata
aatctggagccggtgagcgtgggtctcgcggtatcattgcagcactggggccagatggtaagccctcccgtatcgtagttatctacac
gacggggagtcaggcaactatggatgaacgaaatagacagatcgctgagataggtgcctcactgattaagcattggtaactgtcag
accaagtttactcatatatactttagattgatttacgcgccctgtagcggcgcattaagcgcggcgggtgtggtggttacgcgcagcgt
gaccgctacacttgccagcgccctagcgcccgctcctttcgctttcttcccttcctttctcgccacgttcgccggctttccccgtcaagct
ctaaatcgggggctccctttagggttccgatttagtgctttacggcacctcgaccccaaaaaacttgatttgggtgatggttcacgtag
tgggccatcgccctgatagacggtttttcgccctttgacgttggagtccacgttctttaatagtggactcttgttccaaacttgaacaac
actcaaccctatctcgggctattcttttgatttataagggattttgccgatttcggcctattggttaaaaaatgagctgatttaacaaaa
atttaacgcgaattttaacaaaatattaacgtttacaatttaaaaggatctaggtgaagatcctttttgataatctcatgaccaaaatc
ccttaacgtgagttttcgttccactgagcgtcagaccccgtagaaaagatcaaaggatcttcttgagatcctttttttctgcgcgtaatc
tgctgcttgcaaacaaaaaaaccaccgctaccagcggtggtttgtttgccggatcaagagctaccaactctttttccgaaggtaactg
gcttcagcagagcgcagataccaaatactgtccttctagtgtagccgtagttaggccaccacttcaagaactctgtagcaccgcctac
atacctcgctctgctaatcctgttaccagtcaggcatttgagaagcacacggtcacactgcttccggtagtcaataaaccggtaaacc
agcaatagacataagcggctatttaacgaccctgccctgaaccgacgaccgggtcgaatttgctttcgaatttctgccattcatccgct
tattatcacttattcaggcgtagcaccaggcgtttaagggcaccaataactgccttaaaaaaattacgccccgccctgccactcatcg
cagtactgttgtaattcattaagcattctgccgacatggaagccatcacagacggcatgatgaacctgaatcgccagcggcatcagc
accttgtcgccttgcgtataatatttgcccatggtgaaaacgggggcgaagaagttgtccatattggccacgtttaaatcaaaactgg
tgaaactcacccagggattggctgagacgaaaaacatattctcaataaaccctttagggaaataggccaggttttcaccgtaacacg
ccacatcttgcgaatatatgtgtagaaactgccggaaatcgtcgtggtattcactccagagcgatgaaaacgtttcagtttgctcatg
gaaaacggtgtaacaagggtgaacactatcccatatcaccagctcaccgtctttcattgccatacggaattccggatgagcattcatc
aggcgggcaagaatgtgaataaaggccggataaaacttgtgcttatttttctttacggtctttaaaaaggccgtaatatccagctgaa
cggtctggttataggtacattgagcaactgactgaaatgcctcaaaatgttctttacgatgccattgggatatatcaacggtggtatat
ccagtgatttttttctccattttagcttccttagctcctgaaaatctcgataactcaaaaaatacgcccggtagtgatcttatttcattatg
gtgaaagttggaacctcttacgtgccgatcaacgtctcattttcgccaaaagttggcccagggcttcccggtatcaacagggacacca
ggatttatttattctgcgaagtgatcttccgtcacaggtatttattcggcgcaaagtgcgtcgggtgatgctgccaacttactgatttag
tgtatgatggtgtttttgaggtgctccagtggcttctgtttctatcagctgtccctcctgttcagctactgacggggtggtgcgtaacggc
aaaagcaccgccggacatcagcgctagcggagtgtatactggcttactatgttggcactgatgagggtgtcagtgaagtgcttcatgt
ggcaggagaaaaaaggctgcaccggtgcgtcagcagaatatgtgatacaggatatattccgcttcctcgctcactgactcgctacgc
tcggtcgttcgactgcggcgagcggaaatggcttacgaacggggcggagatttcctggaagatgccaggaagatacttaacaggga

agtgagagggccgcggcaaagccgtttttccataggctccgcccccctgacaagcatcacgaaatctgacgctcaaatcagtggtgg
cgaaacccgacaggactataaagataccaggcgtttccccctggcggctccctcgtgcgctctcctgttcctgcctttcggtttaccgg
tgtcattccgctgttatggccgcgtttgtctcattccacgcctgacactcagttccgggtaggcagttcgctccaagctggactgtatgc
acgaaccccccgttcagtccgaccgctgcgccttatccggtaactatcgtcttgagtccaacccggaaagacatgcaaaagcaccac
tggcagcagccactggtaattgatttagaggagttagtcttgaagtcatgcgccggttaaggctaaactgaaaggacaagttttggtg
actgcgctcctccaagccagttacctcggttcaaagagttggtagctcagagaaccttcgaaaaaccgccctgcaaggcggttttttc
gttttcagagcaagagattacgcgcagaccaaaacgatctcaagaagatcatcttattaatcagataaaatatttgctcatgagccc
gaagtggcgagcccgatcttccccatcggtgatgtcggcgatataggcgccagcaaccgcacctgtggcgccggtgatgccggcca
cgatgcgtccggcgtagaggatctgctcatgtttgacagcttatc

green: Lpp; blue: OpmA; pink: restriction enzyme digestion site (Xba1, Nhe1 and HindIII); yellow: T7Sav

5. Expression of streptavidin displayed on *E. coli* surface

Modified Studier5052 medium: 1x M-stock (25 mM Na_2HPO_4, 25 mM KH_2PO_4, 50 mM NH_4Cl, 5 mM Na_2SO_4) + $MgSO_4$ (2 mM) + glycerol (0.5%) + tryptone (15 g/L) + yeast extract (10 g/L).

A preculture of *E. coli* TOP10(DE3) cells containing the pBAD33-Lpp-OmpA-Sav$_{(codon\ optimized)}$ plasmid was prepared in LB-medium (34 µg/ml chloramphenicol) and incubated overnight at 37°C and 210 rpm in a 50 mL Falcon tube. The modified Studier5052 medium (50 Ml, 34 µg/ml chloramphenicol) was inoculated with 1.6 mL overnight culture in a 250 mL baffled shake flask. The main culture was incubated (37°C, 210 rpm shaking) until an OD_{600} of 0.4 – 1.8 was obtained (~3 h). The Sav expression was induced by addition of 0.2 % L-arabinose and incubation for 4 h at 25°C and 210 rpm.

6. Labeling of surface-displayed streptavidin with a fluorescent antibody

Upon Sav expression on the *E. coli* outer membrane, the cell density was normalized to OD_{600} = 1 in 0.1 mL. The cells were spun down (14.000 g, 30 s) and the supernatant was discarded. Pellets were resuspended in 1 mL PBS buffer (pH 7.4), centrifuged (14.000 g, 30 s) and the supernatant discarded. Pellets were resuspended in 100 µL solution of primary mouse-anti-Sav antibody (ab10020, Abcam) and incubated at room temperature for 30 min. The samples were centrifuged (14.000 g, 30 s) and the supernatant was discarded. The pellets were washed in 1 mL PBS buffer (pH 7.4). The pellets were resuspended in the presence of 100 µL secondary fluorescent (FITC) goat-anti-mouse antibody (F-2761, Thermo Fisher) and incubated on ice for 20 min. The samples were centrifuged (14.000 g, 30 s) and the supernatant discarded. The pellets were washed 1x in 1 mL ice-cold PBS buffer (pH 7.4) before they were resuspended in the same buffer and analyzed by: i) flow cytometry (Attune NxT, Thermo Fisher Scientific, FITC channel: blue laser at 488 nm; Figure 2a) and ii) fluorescence microscopy (Olympus IX81; bright field channel: exposure time: 105 ms, gain: 4.8; FITC channel (488 nm): exposure time: 1 s, gain: 8.6; Figure 2b). As negative controls, the following cell samples and plasmids were used: i) empty vector: Top10(DE3) + pBAD33_empty_vector, ii) cytoplasmatic Sav expression: BL21(DE3) + pET11b_Sav (induction with 50 µM IPTG)[6], iii) periplasmatic Sav expression: Top10(DE3) + pBAD33_ompA-Sav.[4]

7. Labeling of surface-displayed streptavidin with Biot-ATTO565

Upon Sav expression on the *E. coli* outer membrane, the cell density was adjusted to OD_{600} = 2. A volume of 250 µL cells resuspended in PBS buffer (pH 7.4) was centrifuged (14.000 g, 30 s) and the supernatant discarded. Next, the pellets were resuspended in 250 µL Biot-ATTO565 buffer (2 µM final concentration in PBS buffer (pH 7.4); ATTO-TEC GmbH) and incubated on ice for 30 min. The samples were washed 2x in 250 µL PBS buffer before resuspension of the pellets in PBS buffer at an OD_{600} of 0.05. Cellular fluorescence was determined using flow cytometry (Attune NxT, Thermo Fisher Scientific, yellow-green channel: 561 nm; Figure S16).

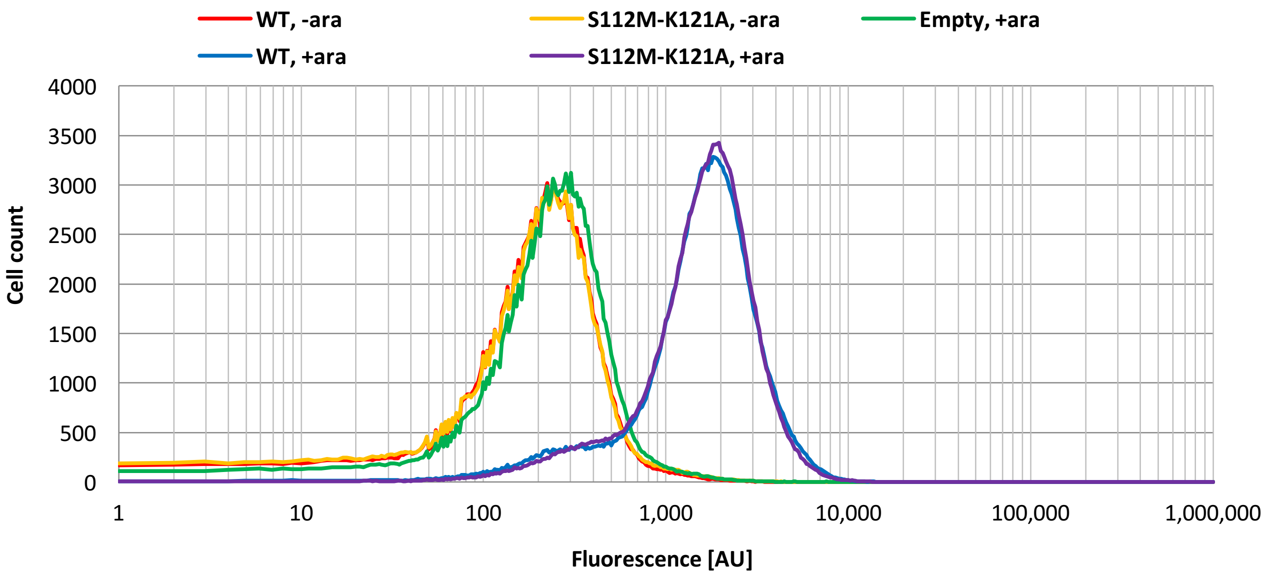

Figure S16: Biotin-ATTO565 staining of *E. coli* cells expressing surface-displayed streptavidin (ara = L-arabinose).

8. Determination of the expression yield of surface-displayed Sav

The expression of Sav mutants on the *E. coli* surface was carried out in a 96-well plate according to section 10. Upon expression, 25 µL cells (OD 2-4) were transferred into a fresh 96-well plate. The plate was centrifuged (3.200 g, 7 min, 4 °C) and the supernatant discarded. The cell pellets were resuspended in 250 µL PBS buffer (50 mM sodium phosphate, 0.9 % NaCl, pH 7.4, OD 0.2-0.4), centrifuged (3.200 g, 7 min, 4 °C) and the supernatant was discarded. Next, the pellets were resuspended in 250 µL of an Biot-ATTO565 solution (0.2 µM in PBS). The cells were incubated for 30 min on ice. After centrifugation, the pellets were resuspended in 250 µL ice-cold PBS and centrifuged. The pellets were resuspended in 250 µL ice-cold PBS for a second time and centrifuged. Finally, the pellets were resuspended in 1 mL ice-cold PBS and the cellular fluorescence was determined by flow cytometry (Attune NxT, Thermo Fisher Scientific, yellow-green channel: 561 nm, Figure S17).

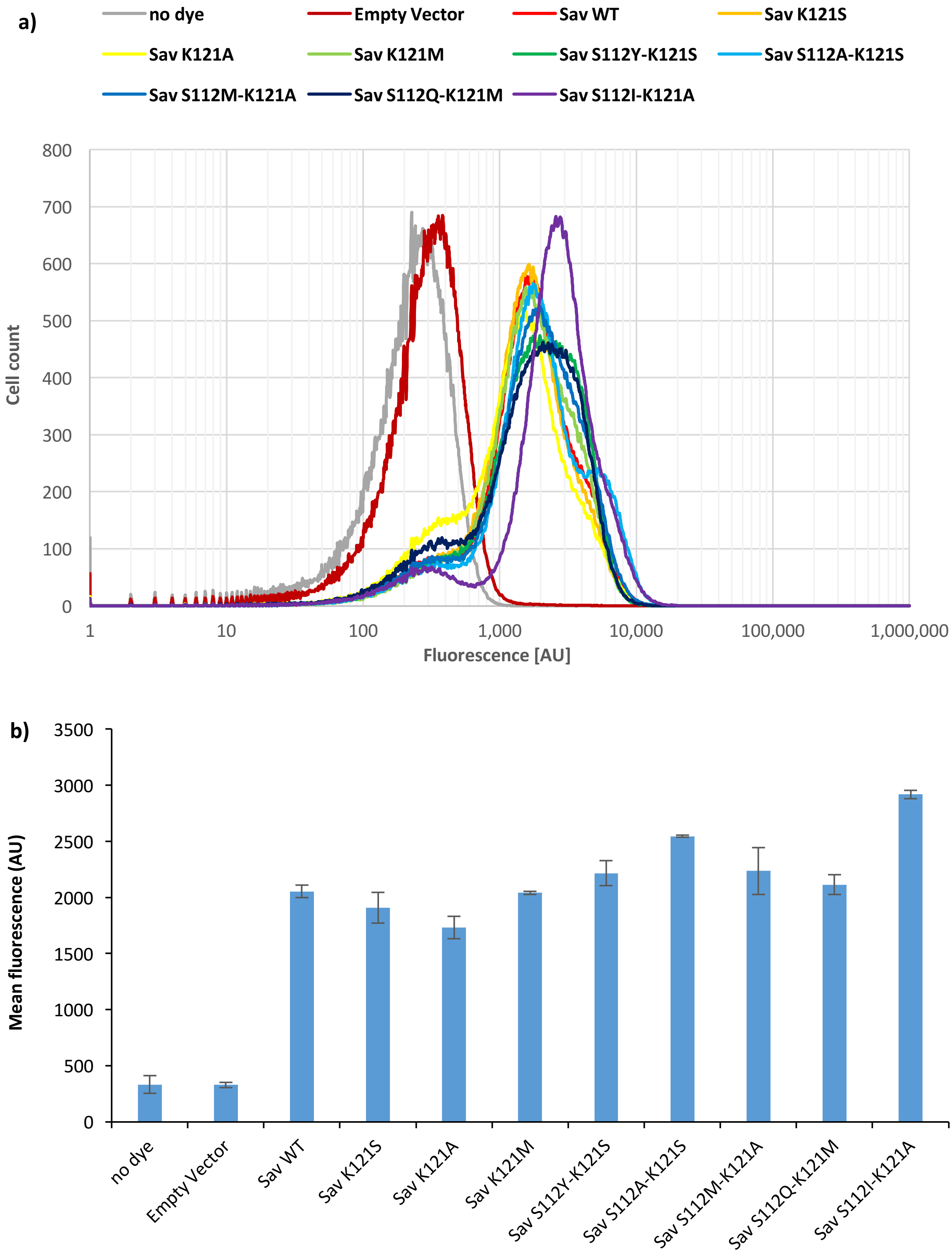

Figure S17: a) Flow cytometry data and b) bar-chart representation of the mean fluorescence of *E. coli* cells upon expression of surface-displayed Sav isoforms labelled with the Biotin-Atto565 dye.

9. Creation of site-saturation mutagenesis libraries using the 22-codon trick

Library generation

The site-saturation mutagenesis libraries were generated using the „22-codon trick" PCR.[7] The PCR was carried out using the conditions collected in Table S5 and Table S6 in the presence of plasmid pBAD33_Lpp-OmpA-Sav$_{WT(codon_omptimized)}$ (Table S4) and the primer mixtures of 12:9:1 of (degenerate) codons NDT, VHG or TGG (forward primers) in the presence of the corresponding reverse primers (Table S7). The PCR products were: i) analyzed by agarose gel electrophoreses, ii) demethylated by addition of 1 µL Dpn1 (NEB) and incubation for 2 h at 37 °C, iii) transformed into chemically competent Top10(DE3) cells by heat shock treatment and iv) transferred on LB-agar$_{cam}$ plates (34 µg/ml chloramphenicol; incubation overnight at 37°C).

Library evaluation

From each library 13 colonies were picked and subjected to Sanger sequencing to assess the library mutation rate. Typically, the codon redundancy within each library was $\leq$ 3. For the Sav S112X-K121A library, the codon redundancy was > 3. Thus, the 96 colonies resulting from the "22 codon trick" were sequenced. The missing mutants were produced using the defined primers reported in Mallin *et al.*[5] and sequenced.

Table S5: General PCR conditions used for the "22-codon trick" site-saturation mutagenesis libraries.

**Reagents**		**1 PCR reaction (µL)**
Q5 buffer, 5x		5
dNTPs (10 mM)		0.5
Forward Primer (1 µM)	NDT	2.72
	VHG	2.05
	TGG	0.23
Reverse Primer (1 µM)	AHN	2.72
	CDB	2.05
	CCA	0.23
Plasmid prep template (25 ng/µL)		1
Q5 Hot Start (2 U/µL)		0.25
ddH_2O		6.25
DMSO (8% final)		2
Total volume		25

Table S6: General PCR thermocycling protocol.

Initial denaturation		95 °C	2 min
25 cycles	Denaturation	95 °C	30 s
	Annealing	70 °C	15 s
	Extension	72 °C	5 min
Elongation		72 °C	10 min
Hold		8 °C	

Table S7: Primers used for site-directed mutagenesis and for the “22-codon trick” site-saturation mutagenesis library generation.

Primer	Sequence
NheI-Lpp-OmpA_fw	GCGACCGGAG**CTAG**CGACAAAGATTGCG
NheI-Lpp-OmpA_rv	CTTTGTCG**CTAG**CTCCGGTCGCAAT
S112X_fw	GCTGACC**AGT**GGCACCACCGAAGC
S112X_rv	GGTGCC**ACT**GGTCAGCAGCCACTGG
K121X_fw	GCCTGG**AAA**AGCACCCTGGTTGGTCATGAT
K121X_rv	GGTGCT**TTT**CCAGGCATTTGCTTCGGTGGTGCC

*Red codons highlight position subjected to the “22-codon trick” mutagenesis.[7] For this purpose, the primer libraries were applied consisting of two degenerate codons (forward: NDT, VHG; reverse: AHN, CDB) plus the tryptophan codon (forward: TGG, reverse: CCA).

Glycerol stocks of “22-codon trick” libraries in 96-well plates

In total, 90 colonies from each library were picked and transferred into a 96-deep well plate containing 300 µL LB_{cam} (34 mg/mL). The column six of the plate was used for control samples: sterile control (A6, B6), pBAD33_empty (C6, D6) and pBAD33_Lpp-OmpA-$Sav_{WT(codon\_optimized)}$ (E6, F6). After the overnight incubation (37 °C, 280 rpm), 100 µL of the overnight culture was mixed with 100 µL 60% glycerol in a sterile microtiter plate and the plate was frozen at -80°C.

10. 96-well plate activity assay using *E. coli* surface display of streptavidin isoforms

The 96-well plate assay was carried out following a modified protocol described by Jeschek *et al.*[4]

Stock solutions

- PBS buffer: 50 mM sodium phosphate, 0.9% NaCl, pH 7.4
- Cofactor **3** buffer (for one 96-well plate): 25 mL PBS buffer (pH 7.4) and 25 µL cofactor stock solution (2 mM; 60 µL Ligand **10** (5 mM), 60 µL Ru-precursor $[CpRu(CH_3CN)_3]PF_6$ (5 mM) and 30 µL DMF)) were mixed.
- Substrate **1** buffer: 4.25 mg substrate **1** (0.5 mM final concentration) were dissolved in 25 mL PBS buffer.

Protein expression

In a 96-deep well plate, 300 µL of LB-medium (34 µg/mL chloramphenicol) were inoculated with fresh colonies (or glycerol stocks) of Sav cells (Top10(DE3) + pBAD33_Lpp-OmpA-$Sav_{(codon\_optimized)}$). After incubation overnight (37 °C, 260 rpm), 240 µL modified Studier5052 medium (34 µg/mL chloramphenicol) were inoculated with 10 µL overnight culture in a fresh 96-deep well plate. Cultures were incubated at 37 °C for 4 h at 310 rpm followed by addition of 10 µL of a 5.2 % L-arabinose solution (0.2 % final concentration) and incubation for 4 h at 25 °C and 310 rpm.

Cofactor loading

The plate was centrifuged (7 min, 4°C, 3.200 g) and the supernatant discarded. The pellets were resuspended in 250 µL ice-cold PBS buffer pH 7.4, the plate centrifuged (7 min, 4°C, 3.200 g), the supernatant discarded and the pellets were resuspended in 250 µL cofactor buffer (2 µM final concentration in PBS buffer, pH 7.4). The cells were incubated on ice for 30 min. Finally, the plate was centrifuged (7 min, 4°C, 3.200 g), the supernatant was discarded and the cell pellets were washed 2x in 250 µL ice-cold PBS buffer (pH 7.4).

Catalysis

The pellets were resuspended in 250 µL substrate buffer (500 µM final concentration), the 96-deep well plate covered with a porous cover slide and incubated at 30 °C, 310 rpm for 16 h.

OD_{600} and fluorescence determination

The OD_{600} (190 µl PBS-buffer + 10 µl reaction mixture) and the fluorescence of aminocoumarin **2** (245 µl PBS-buffer (pH 7.4) + 5 µl reaction mixture, $\lambda_{ex.}$ = 395 nm, $\lambda_{em.}$ = 460 nm) was determined using a TECAN plate reader (Figure S18).

Coefficient of variation

The variation of OD_{600} of a "22-codon trick" Sav library including controls (96 samples total) was determined to be ~ 9 %. The coefficient of variation of ADAse activity measured for a plate containing 96 replicates of cells with Lpp-OmpA-$Sav_{WT(codon\_optimized)}$ was 9 %.

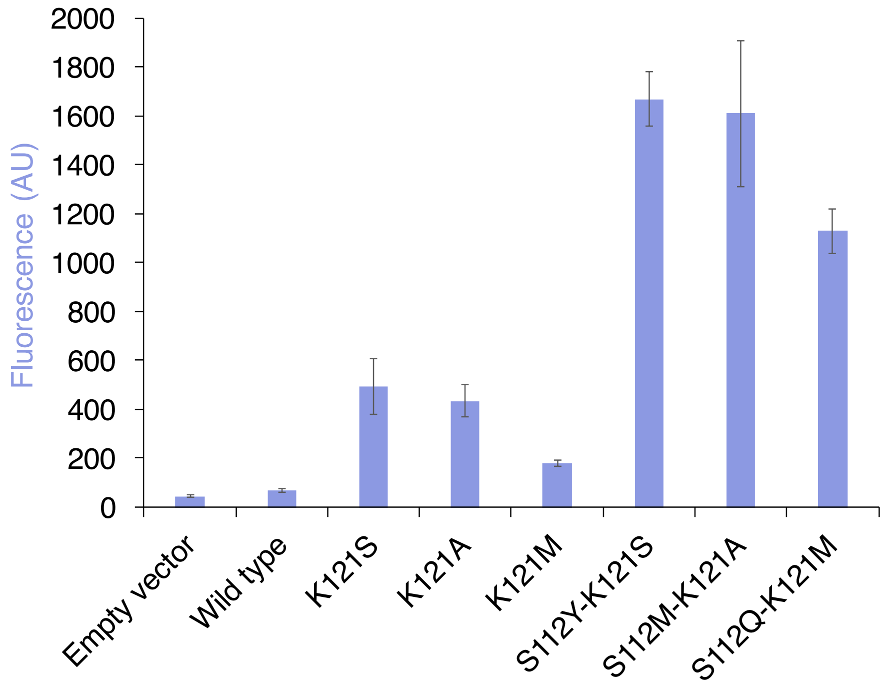

Figure S18: Summary of results from the directed evolution of ADAses on *E. coli´s* surface.

11. *E. coli* cell survival during surface-display catalysis

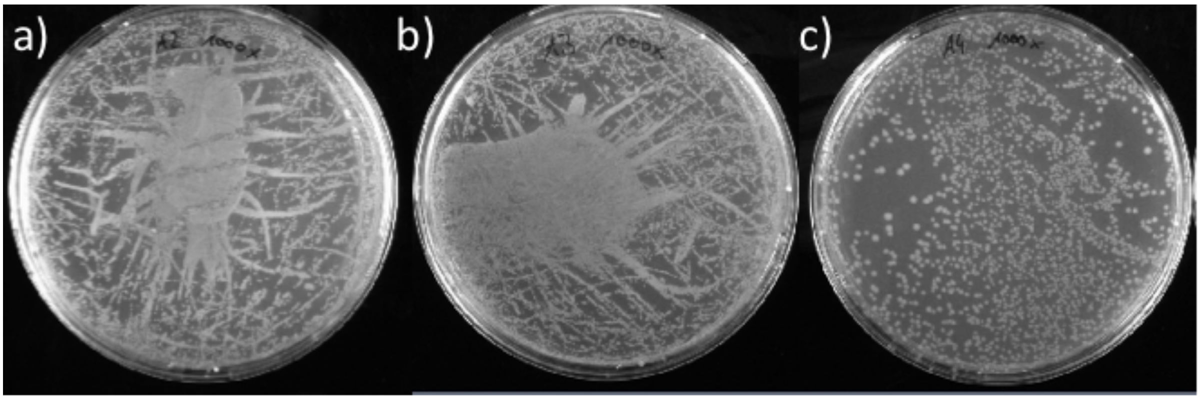

Figure S19: *E. coli* cells survive surface catalysis. (a) Vector pBAD_empty + substrate **1**, (b) Vector pBAD_empty + cofactor **3** + substrate **1**, (c) Vector pBAD_Lpp-OmpA-Sav$_{\mathrm{WT(codon\ optimized)}}$ + cofactor **3** + substrate **1**. Cells were incubated with 2 µM cofactor **3** before initiating the reaction as described above. After 16 h incubation, the cells in the reaction mixture (OD ~2-6) were: i) normalized to OD = 2 by centrifugation of the culture and resuspension in 250 µL PBS buffer, ii) centrifuged and resuspended in 250 µL ice-cold PBS buffer, iii) diluted 1000-fold in PBS buffer and iv) transferred on LB-agar$_{\mathrm{cam}}$ plates and incubated over night at 37°C.

12. Investigation of the potential of biotinylated cofactor **3** to accumulate inside cells

E. coli cell cultivation, expression of surface-displayed Sav, cofactor **3** uptake and reaction was carried out as described in section 10. Cells were incubated with cofactor **3** for 10, 30, 60 and 120 min (Figure S20).

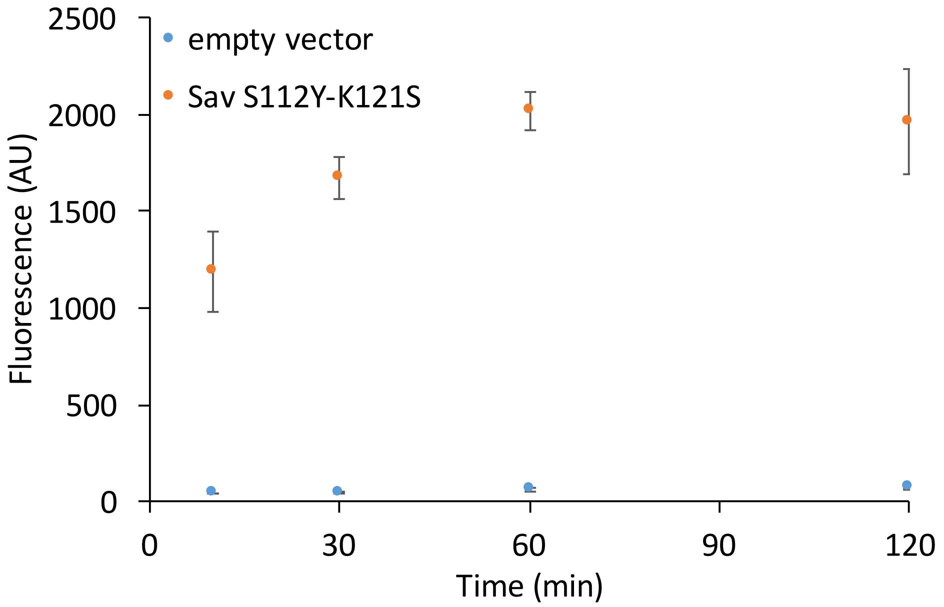

Figure S20. On-cell activity of empty vector and Lpp-OmpA-Sav$_{S112Y-K121S}$ cells upon various incubation times with cofactor **3**.

13. Expression and purification of streptavidin mutants

Sav isoforms were expressed in BL21(DE3) cells containing pET11b_Sav plasmids and purified as described elsewhere.[6]

14. Crystal structure determination of [CpRu(QA-Biot)(H_2O)] **3** · Sav-S112M-K121A

Crystallization of apo-Sav mutant S112M-K121A

Apo-Sav S112M-K121A crystals were obtained at 20°C within two days using the hanging-drop vapor diffusion technique, by mixing 2.0 µL crystallization buffer (2.0 M ammonium sulfate, 0.1 M sodium acetate, pH 4.0) and 3.0 µL protein solution (26 mg/mL lyophilized protein in water). The droplet was equilibrated against a reservoir solution of 100 µL crystallization buffer.

Soaking apo-Sav S112M-K121A crystals with complex [CpRu(QA-Biot)(H_2O)] 3

Single crystals of Sav were soaked for 1 day at 20 °C in a soaking buffer, which was prepared by mixing 0.5 µL of a 7.5 mM stock solution of complex [CpRu(QA-Biot)(H_2O)] **3** (50 % DMSO), 4.5 µL crystallization buffer (2.6 M ammonium sulfate, 0.1 M sodium acetate, pH 4.0), and 0.25 µL of the original protein solution. After the soaking overnight, the crystals were transferred for 10 seconds into a cryo-protectant solution (30 % (v/v) glycerol in crystallization buffer). Next, the crystals were shock-frozen in liquid nitrogen.

Data Processing

The X-ray diffraction data were collected at the Swiss Light Source (SLS) beamline PXIII at a wavelength of 1 Å, processed with software XDS[8] and scaled with AIMLESS (CCP4 Suite)[9]. The structure was solved by molecular replacement using program PHASER (CCP4 Suite)[10] and the structure 2QCB from the PDB as input model with ligand and water molecules removed. For structure refinement REFMAC5 (CCP4 Suite)[10] was used. To model the complex [CpRu(QA-Biot)(H_2O)] **3** into the electron density 3D coordinates of the corresponding non-biotinylated small molecule complex (CSD identifier LILYAI) were imported from the Cambridge Structural Database. For water picking, electron density, and structure visualization, the software COOT[11] was used. Figures were generated with PyMOL (the PyMOL Molecular Graphics System, Version 1.5.0.5, Schrödinger, LLC). Crystallographic details, processing and refinement statistics are given in Table S8.

Structure Refinement

Overall Structures: Apo-crystals of Sav mutants soaked with [CpRu(QA-Biot)(H_2O)] **3** constituted space group $I4_122$ with unit cell parameters collected in Table S8. A single Sav monomer was obtained per asymmetric unit after molecular replacement. The protein residues 2-9 and 135-159 of the *N*- and *C*-terminus, respectively, were not resolved in the electron density, presumably due to disorder. Starting from the Sav monomer, the homotetramer is generated by application of crystallographic C2-symmetry axes along the x-, y- and z-axes of the unit cell (Figure S17). The overall protein structure is virtually identical to the structure of biotin · WT Sav (PDB 1STP, rmsd = 0.76 for all C_α).

Table S8: Crystal-structure processing and refinement statistics.

Sav Mutant	S112M-K121A
PDB code	6FH8
**Data Processing**	
Unit Cell	a, b, c = 57.7 Å, 57.7 Å, 183.6 Å; α, β, γ = 90°
Space group	$I4_122$
Resolution (Å)	45.8 – 1.64
Highest resolution shell (Å)	1.66 - 1.64
R_{merge} (%)	6.5 (83.7)*
No. of unique reflections	19391 (743)
Completeness (%)	98.3 (79.3)
Multiplicity	15.7 (5.5)
I/sig(I)	26.9 (1.7)
CC1/2	1.000 (0.588)
**Structure Refinement**	
R_{work}	0.120
R_{free}	0.159
Rmsd. bond length (Å)	0.0296
Rmsd. bond angle (°)	2.3115
No. ligands:<br>Cofactor<br>Water	 1<br>79
B-factor ($Å^2$):<br>Overall protein<br>[Ru(QA-Biot)]	 27.5<br>39.2

* Data in parentheses are for the highest resolution shell.

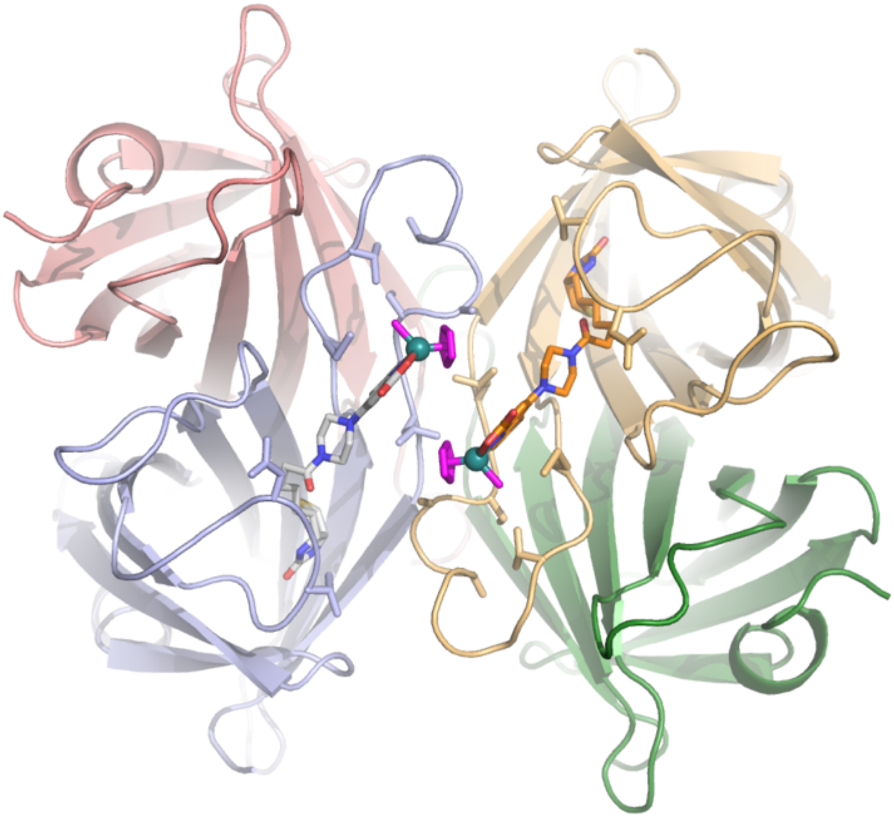

Figure S21: Crystal structure of complex [CpRu(QA-Biot)(H_2O)] **3** · Sav-S112M-K121A (PDB 6FH8). The protein is displayed as cartoon and the cofactor as stick model. The turquoise sphere represents the ruthenium. Magenta atoms were not included in the refinement.

15. Literature

1. Sato, E.; Matsuhisa, A.; Sakashita, M.; Kanaoka, Y., *Chem. Pharm. Bull.*, 1988, **36** (9), 3496-3502.
2. Woronoff, G.; El Harrak, A.; Mayot, E.; Schicke, O.; Miller, O. J.; Soumillion, P.; Griffiths, A. D.; Ryckelynck, M., *Anal. Chem.*, 2011, **83** (8), 2852-2857.
3. Volker, T.; Dempwolff, F.; Graumann, P. L.; Meggers, E., *Angew. Chem., Int. Ed.*, 2014, **53** (39), 10536-10540.
4. Jeschek, M.; Reuter, R.; Heinisch, T.; Trindler, C.; Klehr, J.; Panke, S.; Ward, T. R., *Nature*, 2016, **537** (7622), 661-665.
5. Mallin, H.; Hestericová, M.; Reuter, R.; Ward, T. R., *Nat. Protoc.*, 2016, **11** (5), 835-852.
6. Skander, M.; Humbert, N.; Collot, J.; Gradinaru, J.; Klein, G.; Loosli, A.; Sauser, J.; Zocchi, A.; Gilardoni, F.; Ward, T. R., *J. Am. Chem. Soc.*, 2004, **126** (44), 14411-14418.
7. Kille, S.; Acevedo-Rocha, C. G.; Parra, L. P.; Zhang, Z. G.; Opperman, D. J.; Reetz, M. T.; Acevedo, J. P., *ACS Synt. Biol.*, 2013, **2** (2), 83-92.
8. Kabsch, W., *Acta Crystallogr., Sect. D: Biol. Crystallogr.*, 2010, **66**, 125-132.
9. Evans, P. R., *Acta Crystallogr., Sect. D: Biol. Crystallogr.*, 2011, **67**, 282-292.
10. Murshudov, G. N.; Vagin, A. A.; Dodson, E. J., *Acta Crystallogr., Sect. D: Biol. Crystallogr.*, 1997, **53**, 240-255.
11. Emsley, P.; Cowtan, K., *Acta Crystallogr., Sect. D: Biol. Crystallogr.*, 2004, **60**, 2126-2132.
